# A systematic review of acute and emergency care interventions for adolescents and adults with severe acute respiratory infections including COVID-19 in low- and middle-income countries

**DOI:** 10.7189/jogh.12.05039

**Published:** 2022-11-08

**Authors:** Stephanie Chow Garbern, Pryanka Relan, Gerard M O’Reilly, Corey B Bills, Megan Schultz, Indi Trehan, Sean M Kivlehan, Torben K Becker

**Affiliations:** 1 Department of Emergency Medicine, Warren Alpert Medical School of Brown University, Providence, Rhode Island, USA; 2 Department of Emergency Medicine, Emory Healthcare Network, Atlanta, Georgia, USA; 3 Emergency and Trauma Centre, The Alfred, Melbourne, Australia; 4 School of Public Health and Preventive Medicine, Monash University, Melbourne, Australia; 5 Department of Emergency Medicine, University of Colorado School of Medicine, Aurora, Colorado, USA; 6 Department of Pediatrics, Medical College of Wisconsin, Milwaukee, Wisconsin, USA; 7 Departments of Pediatrics, Global Health, and Epidemiology, University of Washington, Seattle, Washington, USA; 8 Department of Emergency Medicine, Brigham and Women's Hospital, Boston, Massachusetts; 9 Harvard Humanitarian Initiative, Cambridge, Massachusetts, USA; 10 Department of Emergency Medicine, University of Florida, Gainesville, Florida, USA

## Abstract

**Background:**

Severe acute respiratory infections (SARIs) remain a leading cause of death globally, particularly in low- and middle-income countries (LMICs). Early intervention is critical, considering the potential for rapid decompensation in patients with SARIs. We aimed to evaluate the impact of acute and emergency care interventions on improving clinical outcomes in patients >10 years old with SARIs in LMICs.

**Methods:**

A systematic literature search was performed in PubMed, Global Health, and Global Index Medicus databases to identify peer-reviewed studies containing SARI, LMICs, and emergency care interventions. Studies published prior to November 2020 focusing on patients >10 years old were included. A narrative synthesis was performed due to the heterogeneity of identified articles. Risk of bias was assessed using the Risk of Bias 2 and Risk of Bias In Non-Randomized Studies of Interventions tools.

**Results:**

20 223 studies were screened and 58 met the inclusion criteria. Thirty-four studies focused on coronavirus-2019 (COVID-19), 15 on pneumonia, seven on influenza, one study on severe acute respiratory syndrome, and one on undifferentiated SARI. Few COVID-19 studies found a benefit of the tested intervention on clinical status, mortality, or hospital length-of-stay. Little to no benefit was found for azithromycin, convalescent plasma, or zinc, and potential harm was found for hydroxychloroquine/chloroquine. There was mixed evidence for immunomodulators, traditional Chinese medicine, and corticosteroids among COVID-19 studies, with notable confounding due to a lack of consistency of control group treatments. Neuraminidase inhibitor antivirals for influenza had the highest quality of evidence for shortening symptom duration and decreasing disease severity.

**Conclusions:**

We found few interventions for SARIs in LMICs with have high-quality evidence for improving clinical outcomes. None of the included studies evaluated non-pharmacologic interventions or were conducted in low-income countries. Further studies evaluating the impact of antivirals, immunomodulators, corticosteroids, and non-pharmacologic interventions for SARIs in LMICs are urgently needed.

**Registration:**

PROSPERO registration number: CRD42020216117

Severe acute respiratory infections (SARI), including those resulting in pneumonia, cause an estimated four million deaths annually and are the leading communicable cause of death worldwide [1]. While SARIs affect all age groups, the highest mortality is seen in the youngest and oldest age groups, with 45% of all global deaths from lower respiratory infections occurring in those over 70 years old [2-4]. Furthermore, adult and pediatric populations with SARIs have been shown to have substantial differences in epidemiology, clinical presentation, management, and mortality risk [2]. Among adults, older age has been most strongly associated with an increased risk of hospitalization and death from SARIs [3,4].

The 2011 World Health Organization (WHO) case definition of SARI as “an acute respiratory infection with cough and fever which requires hospitalization” was created in response to the 2009-2010 H1N1 influenza pandemic and has since played an important role in public health surveillance and preparedness for monitoring influenza and novel respiratory pathogens [5,6]. While SARIs are a major cause of global morbidity and mortality worldwide, they particularly affect low- and middle-income countries (LMICs) with limited health care resources, especially during outbreaks [7]. SARIs encompass acute respiratory infections regardless of cause, including influenza, respiratory syncytial viruses, coronaviruses, and bacterial and fungal pathogens [5,8]. Certain viral aetiologies of SARI pose a danger of becoming pandemics, such as pandemic influenza A (H1N1/09), Middle East respiratory syndrome coronavirus (MERS-CoV) and most recently, severe acute respiratory syndrome coronavirus 2 (SARS-CoV-2) [9].

The SARI case definition was intentionally developed to be easily applicable and have high sensitivity to capture the burden of influenza and novel respiratory pathogens across a wide variety of settings [5,8]. Although SARIs are a major cause of morbidity and mortality in LMICs, the aetiology of SARIs is often unconfirmed in low-resource settings due to a lack of diagnostic testing capacity [10]. Moreover, the health care resources needed to manage SARIs may be significantly limited in LMICs. Resources such as emergency and critical care support, including supplemental oxygen, antivirals, ventilators, immunomodulators and other therapeutics can impact the course of disease in patients with SARIs and prevent progression to respiratory failure, acute respiratory distress syndrome (ARDS), sepsis, or death, however the availability of these resources and their impact on clinical course are poorly characterized in LMICs [11,12].

Given the potential rapid clinical progression and high mortality rates from SARIs, acute and emergency care interventions are key in improving patient outcomes. Despite recent advances in characterizing the etiologist, incidence, and therapeutics used for SARIs, especially for the novel coronavirus disease 2019 (COVID-19), knowledge gaps persist in identifying acute and emergency care interventions most effective in improving clinical outcomes in adolescents and adults with SARIs in LMICs. Filling these gaps is critical to ensure that limited resources are optimally targeted towards feasible and effective interventions. The aim of this systematic review was to evaluate the impact of acute and emergency care interventions on improving clinical outcomes of adolescent and adults with SARIs in LMICs.

## METHODS

This systematic review was conducted according to the Preferred Reporting Items for Systematic Reviews and Meta-analyses (PRISMA) guidelines for systematic reviews and developed in collaboration with the Global Emergency Medicine Literature Review (GEMLR) group [13]. The review was registered on PROSPERO (registration number CRD42019151080) on October 29, 2020. The published study protocol is shown in Appendix S1 of the **Online Supplementary Document**. As only published, de-identified data were used, this study was exempt from institutional review board approval.

### Search strategy and data sources

The initial search strategy was developed in PubMed and adapted for the Global Health and Global Index Medicus databases using a combination of controlled vocabulary and text word search terms, combining the concepts of SARI, emergency care interventions, and LMICs (Appendix S2 in **Online Supplementary Document**). Free text terms and standardized MeSH headings/subheadings in the context of Boolean operators and appropriate search term truncation were utilized to optimize sensitivity for relevant literature while minimizing excess search results. The search strategy was optimized via multiple trial searches, verifying that all previously identified relevant studies were included. The reference lists of similar reviews were searched manually to both verify search sensitivity and identify other potentially relevant studies. A manual grey literature search was also performed via advanced Google searches targeting organizations known to publish global emergency care literature. Searches were conducted November 2020 to January 2021 and duplicates removed prior to screening.

### Inclusion criteria

Eligible studies included all randomized controlled trials (RCTs) and observational studies with a comparator group that were conducted in LMICS, included patients with SARI, studied an emergency care intervention, and investigated patient-centric clinical outcomes. These criteria are further defined as follows:

1. The WHO SARI case definition of any acute respiratory infection with a history of fever (or measured fever of ≥ 38°C) and cough with onset within the last 10 days which requires hospitalization [5] had to be fulfilled. SARI was used as the case definition because of its global use in public health surveillance, and to capture the wide array of acute respiratory infections that may require emergency care. Studies were screened based on the SARI criteria themselves (i.e., regardless of whether the term “SARI” itself was used) due to a lack of consistent implementation of SARI surveillance across all LMIC contexts. Using a broad definition was necessary, as the aetiology of severe respiratory infections is rarely determined in LMIC emergency settings, especially during the initial treatment and stabilization of patients [10,14,15].
2. Acute and emergency care interventions were defined as relevant interventions (determined by the co-authors’ clinical experience) initiated in the early period of care (within approximately 24 hours), recognizing that the definition of emergency care may extend beyond this period. As much emergency care in LMICs may occur outside of emergency wards or much inpatient care may be conducted in emergency wards due to overcrowding and boarding, no restrictions on setting were used (i.e., pre-hospital or ambulatory settings, inpatient, emergency, and intensive care units were included) [16,17].
3. Only studies which evaluated patient-centric clinical outcomes were included. Primary outcomes included mortality, need for intensive care unit (ICU) admission, need for mechanical ventilation, hospital length-of-stay, clinical severity, symptom duration, symptom severity. Physiologic parameters and time to viral clearance (negative polymerase chain reaction (PCR) test) were included if relevant to patient-centric outcomes (e.g., ability to be discharged).
4. LMICs were defined using the 2020 World Bank classification [18].

Both paediatric and adult articles were included in the initial screening process. However, given the variations in SARI epidemiology and management between adults and children, the two groups were analysed separately. The present study focused on adolescents (10-19 years) and adults (20 years of age or older) as per the WHO definitions [19]. Articles on paediatric populations will be included in future publications.

### Study selection

Two reviewers independently screened each title and abstract. One author (SCG) screened all titles and abstracts and co-authors (CB, GO, PR, and MS) screened one quarter each, with discrepancies resolved by a third reviewer. The same procedure was followed for full-text screening. Articles were excluded if they were not in English or Spanish (based on language capacity of the co-authors), were irrelevant to the topic (eg, chronic disease management), evaluated non-health related outcomes (eg, economic outcomes), were not undertaken in an LMIC, or only consisted of an abstract.

### Assessment of risk of bias

Two authors independently assessed the included studies for risk of bias using the Cochrane Risk of Bias 2 (RoB 2) tool for randomized trials and the Risk Of Bias in Non-Randomized Studies (ROBINS-I) tool for observational studies [20,21]. Discrepancies between reviewers were resolved by a third author.

### Data extraction and analysis

Two authors independently evaluated each article and extracted data on a standardized form. A third author resolved any discrepancies. Form fields included the author, title, publication date, study country, World Bank country classification, study setting, study design, disease studied, intervention description, primary and secondary outcomes, and limitations. A qualitative analysis and narrative synthesis were undertaken because the criteria for a formal meta-analysis (study methodology, intervention, outcomes) were not met. As nearly all studies evaluated interventions for a specific condition/pathogen, the results were presented in terms of the primary condition studied, and subsequently by intervention category, using the United States Pharmacopeia therapeutic categories (eg, antibiotics, corticosteroids, immunomodulators, nutrients and minerals) [22]. Results are presented with the highest quality of evidence first (RCTs without significant risk of bias), followed by results from observational studies and RCTs with serious bias concerns.

## RESULTS

### Description of included studies

**Figure 1:**
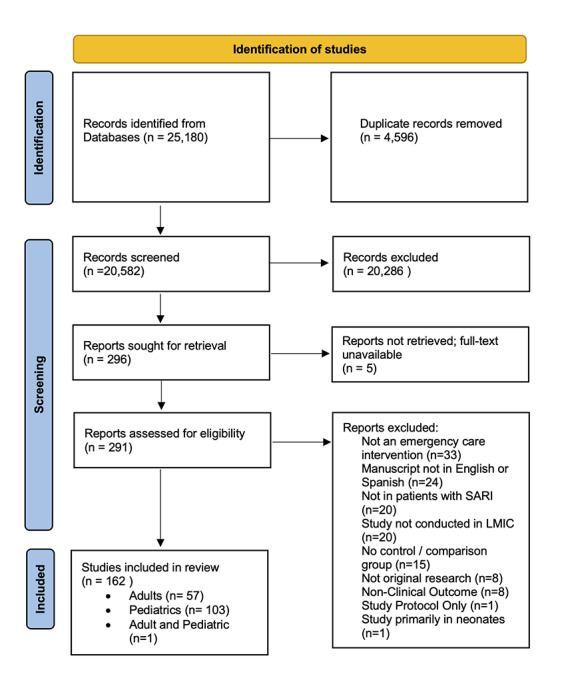
PRISMA flow diagram.

We retrieved 25 180 studies and removed 4957 duplicates. Full texts of 292 articles were screened and 58 studies included for analysis (**Figure 1**). Study characteristics (study country, study design, and types of intervention) are shown in **Table 1**. Thirty-four (59%) studies were on COVID-19, 15 (26%) on community-acquired pneumonia (CAP) or lower respiratory tract infections (LRTI), seven (12%) on influenza (including subtypes H1N1 and H7N9), one on SARS and one on SARI in general. There were 36 (64%) RCTs, 18 (31%) retrospective cohort studies, two (3%) case-control studies, one (2%) non-randomized trial, and one (2%) prospective cohort study. More than two-third of included studies (n = 40; 69%) were conducted in upper middle-income countries while 18 (31%) were in lower middle-income countries. No studies were conducted in low-income countries (LIC). The most represented regions (using WHO region definitions) were Western Pacific (n = 29; 50%), Eastern Mediterranean (n = 15; 25.9%), Americas (n = 10; 17.2%), South-East Asia (n = 3; 5.2%), and Africa (n = 1; 1.7%). Most studies were conducted in China (n = 26; 44.8%), Iran (n = 10; 17.2%), and Brazil (n = 9; 15.5%). A full listing of study countries is shown in **Table 1****.**

**Table 1:** Characteristics of the included studies (n = 58)

Disease/condition	Number of studies	Study design	Countries	Interventions
**COVID-19**	22	RCT	Brazil, China, Egypt, India, Iran	Antibiotics, antiparasitics, antivirals, convalescent plasma, corticosteroids, immunomodulators, nutrients and minerals, respiratory tract agents, and TCM
	1	Non-randomized trial	China	Immunomodulator
	10	Retrospective cohort	China, Iran, Pakistan, Thailand	Antibiotic, antiviral, corticosteroid, antiparasitic, immunomodulator, TCM
	1	Case-control	China	Immunomodulator
**CAP/LRTI**	11	RCT	Brazil, China, Egypt, Iran, Nigeria, Vietnam	Antibiotic regimens, corticosteroids, TCM, nutrients and minerals
	1	Prospective cohort	Malaysia	Antibiotic
	3	Retrospective cohort	Brazil, Malaysia, Pakistan	Antibiotics, corticosteroids, guideline adherence
**Influenza** **(incl. H1N1, H7N9)**	2	RCT	Brazil, China	Antivirals
	4	Retrospective cohort	Brazil, China	Antivirals
	1	Case-control	China	Corticosteroids
**SARS**	1	Retrospective cohort	China	Corticosteroids
**SARI**	1	RCT	Mexico	Antiparasitic

CAP – community-acquired pneumonia, COVID-19 – coronavirus disease 2019, LRTI – lower respiratory tract infection, RCT – randomized-controlled trial, SARS – severe acute respiratory syndrome (SARS-associated coronavirus), SARI – severe acute respiratory infection, TCM – traditional Chinese medicine, N – number of participants

### Risk of bias

**Figure 2:**
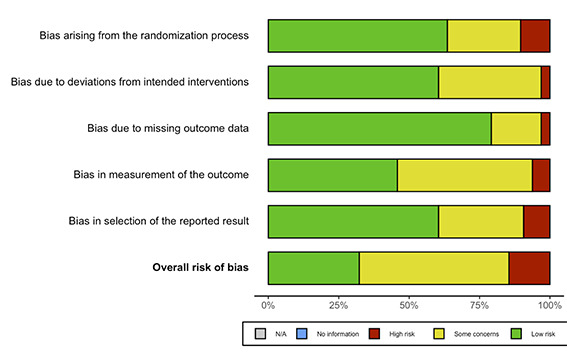
Summary of risk of bias assessments for included randomized-controlled trials using Cochrane’s Risk of Bias tool for randomized trials version 2 (RoB 2).

**Figure 3:**
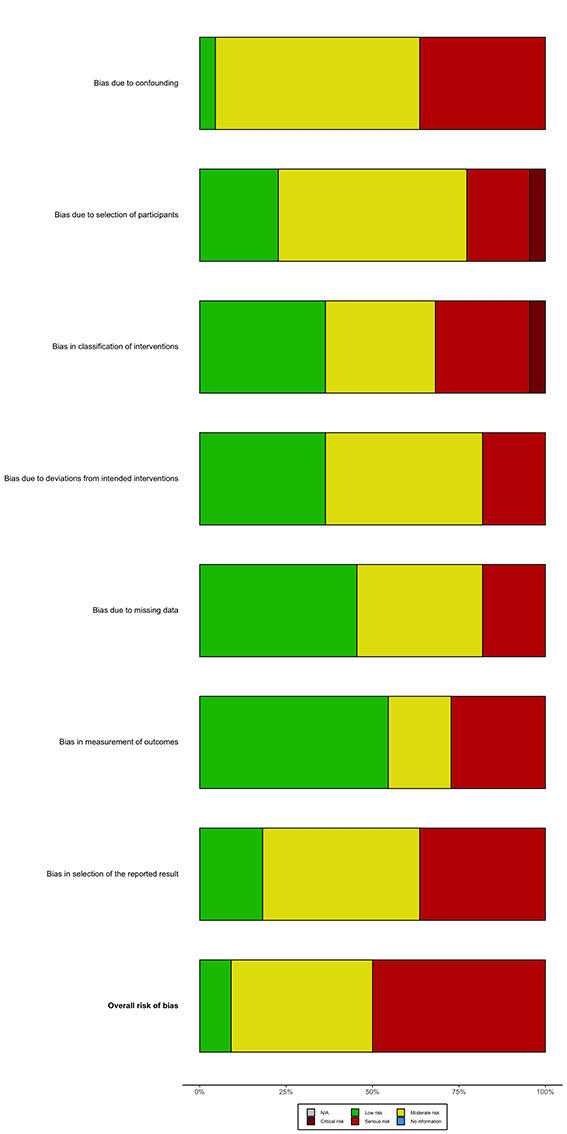
Summary of risk of bias assessments for included non-randomized studies according to the ROBINS-I tool.

**Figure 2** and **Figure 3** summarize the RoB 2 assessment for RCTs and ROBINS-I assessment for non-randomized and observational studies. Figure S1 and S2 in the **Online Supplementary Document** show the individual RoB 2 and ROBINS-I assessments for each study. Among RCTs, eight studies had overall low risk of bias, 23 had some concerns, and five had high risk. Among non-randomized and observational studies, two studies had low overall risk of bias, nine had moderate risk, and 11 had serious risk of bias.

### COVID-19

Of the 34 articles on COVID-19, there were 22 RCTs, one non-randomized trial, and 11 observational studies. Interventions included antibiotics, antiparasitics, antivirals, convalescent plasma, corticosteroids, immunomodulators, nutrients and minerals, respiratory tract agents, and traditional Chinese medicine (TCM) (**Table 2**). Multiple studies used an ordinal clinical status severity scale as a primary outcome, ranging from meeting discharge criteria (lowest severity), to requiring hospitalization with supplemental oxygen (moderate severity), to death (highest severity); an example of one of these scales is shown in Table S3 in the **Online Supplementary Document**. The “standard of care” treatments provided to control groups were notably inconsistent, with most control groups having received treatment according to the national or hospital guidelines for standard of care at the time of the study period.

**Table 2:** Summary of findings on COVID-19 (n = 34 studies)

Study ID	Country	Design	Population	N	Intervention	Outcome	Key findings	RoB
**Antibiotics**								
Furtado, 2020 [23]	Brazil	RCT	≥18 y admitted with suspected or confirmed COVID-19 and at least one additional severity criteria	397	PO 500mg azithromycin once daily for10 d in addition to standard of care vs standard of care (HCQ 400 mg twice daily for 10 d)	Clinical status at 15-d using a six-level ordinal scale; mortality; hospital LOS	No difference in clinical status between the azithromycin and control groups (OR = 1.36, 95% CI = 0.94 to 1.97, *P* = 0.11), death at 29 d (31% vs 30%, *P* = 0.63), or hospital LOS (26 vs 18 d, *P* = 0.064).	Some Concerns
Sekhavati 2020 [24]	Iran	RCT	≥18 y admitted with +COVID-19 PCR test and radiographic findings of COVID pulmonary involvement	111	PO Azithromycin 500mg daily for 5 d in addition to standard of care vs standard of care (oral LPV/r 400/100 mg twice daily and oral HCQ 400 mg daily)	Mortality; Hospital LOS; ICU admission; Vital Signs; QTc prolongation or cardiac arrhythmia	No difference in mortality between groups (*P* = 0.495). Hospital LOS shorter (4.61 d vs 5.96 d; *P* = 0.02), RR lower (15.85 rpm vs .17.42 rpm; *P* = 0.010), and higher SpO2 (93.95%vs92.40%; *P* = 0.030) in treatment vs control group. No patients had cardiac arrhythmia or QTc prolongation.	Some Concerns
**Antiparasitic**								
Abd-Elsalam, 2020 [25]	Egypt	RCT	Patients with confirmed COVID-19 by RT-PCR with mild, moderate, severe disease	194	Hydroxychloroquine 400mg BID day 1 then 200mg BID for 15 d vs standard of care (paracetamol, antibiotics, oseltamivir as needed)	Recovery within 28 d; need for mechanical ventilation; death	No difference in recovery at 28 d (53.6% vs 34%, *P* = 0.06), overall mortality (6.2% vs 5.2%, *P* = 0.77), or need for mechanical ventilation (4.1% vs 5.2%, *P* = 0.75) in HCQ vs control group.	High
Borba, 2020 [26]	Brazil	RCT	≥18 y with COVID-19 and RR>24rpm, SpO2 < 90%, or shock, admitted to hospital	81	High-dose CQ (600mg BID for 10 d, total dose 12g) vs low-dose CQ (450mg BID tapering down Day 1-9, total dose 2.7g)	28-d mortality; QTc prolongation	Greater risk of death in (39% vs 15% (OR = 3.6, 95% CI = 1.2 to 10.6) in high vs low dose groups although no difference when age-adjusted. Greater QTc prolongation 18.9% vs 11.1% and VT 2.7% vs 0% in high vs low-dose group.	Some Concerns
Cavalcanti, 2020 [27]	Brazil	RCT	≥18 y admitted to hospital with suspected or confirmed mild to moderate COVID-19	665	Group 1: Standard care Group 2: Standard care + HCQ 400mg BID Group 3: Standard care + HCQ 400mg BID + Azithromycin 500mg once daily	Clinical status at 15 d, using 6-level ordinal scale	No difference in clinical status at 15 d for either HCQ alone (OR = 1.21; 95% CI = 0.69 to 2.11; *P* = 1.00) or HCQ plus azithromycin (OR = 0.99; 95% CI = 0.57 to 1.73; *P* = 1.00). No difference in hospital LOS, need for MV or in-hospital mortality. Prolongation of the QTc and elevation of liver-enzyme levels were more frequent in patients receiving hydroxychloroquine, alone or with azithromycin, than in those who were not receiving either agent.	Some Concerns
Yu, 2020 [28]	China	Retrospective cohort	≥18 y with COVID-19 admitted to hospital with critical illness	550	PO HCQ 200mg BID for 7-10 d in addition to standard of care (including antivirals, antibiotics, IVIG comparable between groups)	Hospital mortality; hospital LOS	Decreased mortality 18.8% vs 47.4% (*P* < 0.001) in HCQ vs control group. No difference in mean hospital LOS 32 vs 30 d (*P* = 0.314) although longer hospital stay before death 15 vs 8 d (*P* = 0.027).	Serious
**Antiviral**								
Abbaspour Kasgari, 2020 [29]	Iran	RCT	18-80 y with moderate COVID-19	48	PO 400/60mg sofosbuvir/daclatasvir once daily and ribavirin 600mg twice daily vs hydroxychloroquine 400mg single dose with lopinavir/ritonavir 400/100mg BID with or without ribavirin 600mg BID (control)	Hospital LOS; need for ICU admission, mechanical ventilation; mortality	No difference in median length of hospital stay (6 d) in treatment vs control group (*P* = 0.398). No difference in need for ICU admission (*P* = 0.109), mechanical ventilation (*P* = 0.109) or death (*P* = 0.234).	Some Concerns
Deng, 2020 [30]	China	Retrospective cohort	≥18 y laboratory-confirmed COVID-19 admitted with pneumonia	33	PO Arbidol 200mg q8H in addition to lopinavir/ritonavir 400mg/100mg q12h until negative RT-PCR	Conversion to negative RT-PCR at day 7 and 14; pneumonia progression or improvement on chest CT at day 7	Negative RT-PCR combination vs monotherapy was 75% vs 35% (*P* < 0.05) at 7 d; 94% vs 52.9% (*P* < 0.05) at 14 d; improvement on chest CT at 7 d was 69% vs 29% (*P* < 0.05).	Serious
Prasithsirikul, 2020 [31]	Thailand	Retrospective cohort	All patients with COVID-19 confirmed on RT-PCR	41	PO Favipiravir 200mg tablets BID. Day 1: 8 tablets, Day 2-10: 3 tablets	Hospital LOS; time to viral clearance	No significant difference in LOS between treatment vs control (8 vs 10 d; *P* = 0.86). Earlier median time to viral clearance in favipiravir vs control (6 vs 8 d; *P* = 0.11)	Serious
Sadeghi, 2020 [32]	Iran	RCT	≥18 y with moderate or severe COVID-19 admitted to hospital	66	PO 400 mg sofosbuvir and 60 mg daclatasvir + standard care vs standard care alone (hydroxychloroquine 200mg BID with or without lopinavir/ritonavir 200 mg/50 mg BID).	Clinical recovery at 14 d; Hospital LOS; Mortality; Mechanical Ventilation	No difference in clinical recovery at 14 d between treatment vs control group (88% vs 67%, *P* = 0.076). Shorter LOS in treatment vs control (6 (IQR = 4-8) vs 8 (IQR = 5-13) days, *P* = 0.029). No difference in mortality (9% vs 15% *P* = 0.708) or mechanical ventilation (9% vs 21%, *P* = 0.303) between treatment and control.	Low
Wang (Y), 2020 [33]	China	RCT	≥18 y with severe COVID-19 admitted to hospital	237	IV Remdesivir 200mg on Day 1 then 100mg Days 2-10 vs placebo. Concomitant use of lopinavir-ritonavir, interferons, and corticosteroids permitted (non-standardized, authors reported no differences between groups)	Time to clinical improvement defined as decline of two levels on a 6-point ordinal scale of clinical status through Day 28; hospital LOS; mortality	No difference in time to clinical improvement (HR = 1.23 d, 95% CI = 0.87 to 1.75), 28-d mortality (14% vs 13%, diff = 1.1%, 95% CI = -8.1 to 10.3) or hospital LOS (25 vs 24 d, diff 0 (95% CI = -4 to 4) in remdesivir vs control group.	Some Concerns
**Antiviral + antibiotic + steroid**								
Vahedi, 2020 [34]	Iran	Retrospective cohort	≥18 y with laboratory confirmed moderate COVID-19	60	Group 1: Azithromycin 250mg daily, prednisolone 25mg daily, naproxen 250mg BID, and lopinavir/ritonavir 200/50 mg BID vs Group 2: meropenem 1g q8h, levofloxacin 500mg daily vancomycin 1g q12h, hydroxychloroquine 200mg q12h, oseltamivir 75mg BID	Hospital LOS; vital signs; laboratory values at day 3	Decreased LOS in Group 1 with 6.97 d (SD = 3.08) vs 9.93 d (SD = 3.16) in Group 2 (*P* = 0.001). Compared to baseline at Day 3, decrease in CRP in Group 1 (79.5 to 24.0, *P* < 0.001), increase in SpO2 (86.7 to 89.8, *P* = 0.011), decrease in body temperature (37.7 to 37.0, *P* < 0.001), otherwise no differences in RR, heart rate, BP, or other laboratory markers.	Serious
**Convalescent plasma**								
Agarwal, 2020 [35]	India	RCT	≥18 y with moderate COVID-19 confirmed with RT-PCR admitted to hospital	464	200mL convalescent plasma for 2 doses 24 h apart	Composite of progression to severe disease (Pao_2_/FiO2 ratio <100 mm Hg) any time within 28 d of enrolment or all-cause mortality at 28 d	No significant difference in composite outcome (19% vs 18%; RR = 1.04, 95% CI = 0.71 to 1.54). No difference in all-cause mortality (15% vs 14%; RR = 1.04, 95% CI = 0.66 to 1.63) or progression to severe disease (17% in both; RR = 1.04, 95% CI = 0.54 to 1.98) in plasma vs placebo at 28 d.	Some Concerns
Li, 2020 [36]	China	RCT	≥18 y with severe or critical COVID-19 confirmed with RT-PCR admitted to hospital	103	Convalescent plasma 4-13 ml/kg of recipient body weight for single transfusion	Time to clinical improvement within a 28-d period (reduction of 2 points on 6-point disease severity scale); 28-d mortality	No significant difference in clinical improvement within 28 d (51.9% vs 43.1 plasma vs control; HR = 1.4, 95% CI *=* 0.79 to 2.49). No significant difference in 28-d mortality (15.7%vs 24.0% plasma vs control; OR = 0.59; 95% CI = 0.22 for 1.59. Possible benefit in those with severe (non-critical) disease with 91.3% vs 68.2% having clinical improvement at 28 d (HR = 2.15, 95% CI = 1.07 to 4.32; *P* = 0.03).	Some Concerns
**Corticosteroids**								
Edalatifard, 2020 [37]	Iran	RCT	≥18 y with COVID-19 admitted to hospital with hypoxia <90% and elevated CRP and IL-6	68	IV methylprednisolone 250mg daily for 3 d vs standard of care (hydroxychloroquine sulfate, lopinavir and naproxen)	Time to clinical improvement or death; hospital LOS	Reduced median days in hospital (11.62 vs 17.61, *P* = 0.006) and reduced median time to improvement (11.84 vs 16.44 d, *P* = 0.011) and reduced mortality (5.9% vs 42.9%, *P* < 0.001) in treatment vs control.	Some Concerns
Jeronimo, 2020 [38]	Brazil	RCT	≥18 y with COVID-19 admitted to hospital with Sp02 < 95%	393	IV methylprednisolone 0.5mg/kg BID for 5 d	28-d mortality; need for intubation	No significant difference in mortality (37.1% vs 38.2%, *P* = 0.629) or need for intubation (19.4% vs 16.8%, *P* = 0.654) in methylprednisolone vs placebo. In subgroup analysis in patients over 60 y, lower mortality 46.6% vs 61.9%, *P* = 0.039).	Low
Ma, 2020 [39]	China	Retrospective cohort	Adult patients with COVID-19 admitted to hospital	72	Corticosteroid use vs no corticosteroid use. Low-dose: 40mg/d Shock-dose: 80mg/d	Hospital mortality; hospital LOS; time to viral clearance	No significant difference in hospital mortality (4.3% vs 8.0%, *P* = 0.550), LOS (18.7 vs 21.0 d, *P* = 0.212), and time of viral clearance (16.1 vs 19.4 d, *P* = 0.184).	Moderate
Rana, 2020 [40]	Pakistan	Retrospective quasi-experimental	Adult patients admitted to high-dependency unit (HDU)/ICU on BiPAP	60	Dexamethasone 8mg BID vs methylprednisolone 40mg BID	P/F ratio	Greater improvement in P:F in the dexamethasone vs methylprednisolone group (170 vs 118, *P* < 0.001).	Moderate
Tomazini, 2020 [41]	Brazil	RCT	≥18 y with confirmed or suspected COVID-19 infection receiving mechanical ventilation within 48 h of meeting criteria for moderate to severe ARDS	299	IV Dexamethasone 20mg once daily for 5 d, then 10mg once daily for 5 d or until ICU discharge vs standard of care	Ventilator-free days during first 28 d, defined as being alive and free from mechanical ventilation; 28-d mortality	Higher mean number of ventilator-free days (6.6 vs 4.0 d, *P* = 0.04) in dexamethasone vs control group. No significant difference in all-cause mortality at 28-d (56.3% vs 61.5%, HR = 0.97, 95% CI = 0.72 to 1.31, *P* = 0.85).	Low
Wu, 2020 [42]	China	Retrospective cohort	Patients with laboratory-confirmed or clinically diagnosed severe or critical COVID-19 admitted to hospital	1514	IV corticosteroid use	Hospital mortality	No benefit for corticosteroids on in-hospital mortality in either severe cases (HR = 1.77; 95% CI = 1.08 to 2.89; *P* = 0.023), or critical cases (HR = 2.07; 95% CI = 1.08 to 3.98; *P* = 0.028).	Serious
Zha, 2020 [43]	China	Retrospective cohort	Patients with COVID-19 confirmed by RT-PCR admitted to hospital	31	Methylprednisolone 40mg once or twice daily	Viral clearance time; hospital LOS; duration of symptoms	No difference in virus clearance time (HR = 1.26; 95% CI, 0.58-2.74), hospital length of stay (HR = 0.77; 95% CI = 0.33 to 1.78), or duration of symptoms (HR = 0.86; 95% CI = 0.40 to 1.83) in corticosteroid vs non-corticosteroid groups.	Low
**Immunomodulator**								
Cheng, 2020 [44]	China	RCT	≥18 y with COVID-19 confirmed with RT-PCR, pneumonia on imaging, and lymphopenia (PBL cell count of 800 per uL)	200	SQ rhG-CSF, 5 μg/kg, at days 0-2	Time to clinical improvement defined as decline of at least 1 point on a 7-point ordinal scale	No difference in time to clinical improvement between the rhG-CSF group and the usual care groups (median (IQR) = 12 (10-16) vs 13 (11-17) days; HR = 1.28; 95% CI = 0.95 to 1.71).	Some Concerns
Fu, 2020 [45]	China	Non-randomized clinical trial	≥18 y with hospitalized with moderate COVID-19 disease	33	Nasal inhalation TFF2 and IFN-k in water over 20-30 min by nasal mask 3 times every 48 h vs standard of care (included TCM and anti-pyretics)	Safety of aerosol inhalation of IFN-k plus TFF2; Hospital LOS; cough resolution; improvement of CT imaging; rate of negative viral RNA at 10 d	No severe adverse effects in treatment group. Shorter hospital LOS – 12 d (IQR = 7-20) vs 15 d (IQR = 10-25), *P* < 0.001), median time to CT improvement = 5 (IQR = 3-9) vs 8.5 (IQR 3-17) days, *P* < 0.05), median time to viral clearance = 6 (IQR = 2-13) vs 9.5 (IQR = 3-23) days, *P* < 0.05), and median time to cough resolution = 4.5 d (IQR = 2.0-7.0) vs 10.0 d (IQR = 6.0-21.0), *P* < 0.005) in treatment vs control group.	Serious
Gharebaghi, 2020 [46]	Iran	RCT	≥18 y with severe COVID-19 admitted to hospital	59	IV Immunoglobulin (IVIG) 5g daily for 3 d vs placebo	In-hospital mortality	Lower in-hospital mortality rate (20% vs 48.3%, *P* = 0.022) in IVIG vs control group.	Some Concerns
Hao 2020 [47]	China	Case-control	Confirmed SARS-CoV-2-positive hospitalized patients	104	Recombinant human IFN-τ2b spray 100 000 U Q6H for 7 d	Duration of SARS-CoV-2 viral shedding from the respiratory tract; Mechanical Ventilation; ICU admission	No difference in viral shedding time (12 vs 15 d, *P* = 0.21) in IFN vs control group. No difference in hospital LOS (16 vs 21 d, *P* = 0.08) and or need for mechanical ventilation (25% vs 37%, *P* = 0.28) IFN vs control group	Low
Rahmani, 2020 [48]	Iran	RCT	≥18 y with severe COVID-19 pneumonia confirmed by PCR and radiographic imaging	66	SQ IFN τ-1b 250mcg every other day for 2 weeks	Time to clinical improvement defined as improvement of at least 2 points on 6-point ordinal scale; Hospital LOS; 28-d Mortality	Shorter time to clinical improvement - 9 (IQR = 6-10) vs11 (IQR = 9-15) days, *P* = 0.002), shorter hospital LOS – 11 (IQR = 9-13) days in the IFN group vs 13 (IQR = 10-17) days, *P* = 0.05) in treatment vs control group. No difference in 28-d mortality (6.06% vs 18.18% IFN vs control, *P* = 0.12).	Low
Zheng, 2020 [49]	China	Retrospective cohort	18-85 y admitted with COVID-19	181	Tocilizumab 4-8mg/kg first dose. Second dose given if still febrile after 24 h vs conventional treatment (not specified)	Hospital LOS; clinical symptoms and laboratory values	Decreased WBC, CRP, Tmax, minimum SpO2, RR, heart rate post-treatment (*P* < 0.001). Shorter hospital LOS (mean = 16.4 vs 27.5 d, *P* < 0.001) and fewer deaths (1.1% vs 9.8%, *P* = 0.018) in conventional vs tocilizumab group although more patients with critical/severe illness (84.8% vs 46.1%, *P* < 0.001) in tocilizumab vs conventional	Serious
**Nutrients and minerals**								
Abd-Elsalam, 2020 [50]	Egypt	RCT	Patients with confirmed COVID-19 by RT-PCR with mild, moderate, severe, and critical disease	191	Zinc sulfate 220mg BID + hydroxychloroquine 400mg BID on day 1 then 200mg BID for 5 d vs HCQ only	Hospital LOS; clinical recovery at 28 d; mechanical ventilation; mortality	No significant difference in hospital LOS LOS (13.5 vs 14 d, *P* = 0.553), clinical recovery at 28 d (79.2% vs 77.9%, *P* = 0.969), mechanical ventilation (*P* = 0.537) and mortality (94.8% vs 94.7%; *P* = 0.986) between Zinc vs control group	Some Concerns
**Respiratory tract agent**								
Ansarin, 2020 [51]	Iran	RCT	≥18 y with COVID-19 pneumonia admitted to hospital	78	PO bromhexine hydrochloride 8mg Q8H + standard of care. Standard of care included HCQ 200 mg daily	Rate of ICU admission, intubation/mechanical ventilation, and 28-d mortality	Decreased ICU admissions (5.1% vs 28.2%, *P* = 0.006), need for mechanical ventilation (2.6% vs 23.1%, *P* = 0.007) and death (0% vs 12.8%, *P* = 0.027) in the bromhexine vs control group.	Some Concerns
deAlencar 2020 [52]	Brazil	RCT	≥18 y with severe COVID-19 (suspected or confirmed) admitted to hospital	135	IV NAC 14g in first 4 h and 7g in next 16 h vs placebo	Invasive mechanical ventilation; admission to ICU, time in ICU, and mortality	No difference in need for mechanical ventilation between groups (20.6% vs 23.8%, *P* = 0.641). No difference in admission rate to ICU (43% vs 47%; *P* = 0.557) or mortality (14% both, *P* = 0.940)	Low
**Traditional Chinese medicine**								
Liu, 2020 [53]	China	Retrospective cohort	Patients with COVID-19 confirmed by RT-PCR without criteria for critical illness	80	Jinhua Qinggan granules 6g BID for 7 d	Time to viral clearance on RT-PCR from respiratory specimen; improvement on chest CT	Time to viral clearance was 7 ± 4 vs 10 ± 4 d (*P* = 0.010) and pneumonia recovery time indicated by chest CT was 8 ± 4 vs 10 ± 5 d (*P* = 0.021) for treatment vs control group.	Serious
Wang (J), 2020 [54]	China	RCT	Patients with COVID-19 admitted to hospital without respiratory failure requiring mechanical ventilation	47	Keguan-1 19.4g BID with standard of care vs standard of care (alpha interferon inhalation, 50 μg BID and lopinavir/ritonavir, 400mg/100 mg twice daily)	Time to ARDS development; time to fever resolution and recovery of lung injury on CT or radiograph at day 7 and 14	Decreased development of ARDS 4.2% vs 26.1% (*P* = 0.048), shorter mean time to fever resolution 1.5 vs 3.0 d (*P* = 0.035), but no difference in proportion of recovery of lung injury 87.5% vs 69.6% (*P* = 0.168) in treatment vs control group.	High
Xiao, 2020 [55]	China	RCT	Adults aged 18-85 y old with suspected and diagnosed cases of COVID-19 meeting the diagnostic criteria	283	Group 1: Huo Xiang Zhengqi dropping pills 2.6g BID and Lianhua Qingwen 6g q8h and standard of care Group 2: Lianhua Qingwen 6g q8h and standard of care Group 3: standard of care (PO oseltamivir 75mg daily, arbidol 200mg q8h, ribavirin 150mg q8h)	Symptom improvement; progression to “severe status” at 14 d	No significant difference in symptom resolution or progression to severe disease between the three groups (*P* = 0.089).	Some Concerns
Xiong, 2020 [56]	China	RCT	Adults aged 18-75 y admitted with COVID-19 (mild to severe disease)	42	Xuanfei Baidu decoction 200ml BID in addition to standard of care vs standard of care (not specified)	Symptom resolution (cough, fever, fatigue) at 7 d	Higher rate of symptom resolution in fever 90% vs 72.3% (*P* = 0.043), cough 76.5% vs 38.9% (*P* = 0.028), and fatigue 78.9% vs 42.9% (*P* = 0.039) in treatment vs control group.	Some Concerns

ARDS – acute respiratory distress syndrome, BID – twice daily, CI – confidence intervals, CRP – C reactive protein, CT – computed tomography, HCQ – hydroxychloroquine, HR – hazard ratio, ICU – intensive care unit, IFN – interferon, IQR – interquartile range, OR- odds ratio, IV – intravenous, LOS – length of stay, LPV/r – lopinavir/ritonavir, N – number of participants, NAC – N-acetylcysteine, P:F – Pao_2_:FiO2, PO – per oral; QTc – corrected QT interval; rhG-CSF – recombinant human granulocyte colony stimulating factor, RR – respiratory rate, RoB – risk of bias, SpO2 – oxygen saturation, WBC – white blood cells

### Antibiotics

Two studies evaluated the use of antibiotics alone; both were RCTs evaluating azithromycin for hospitalized COVID-19 patients and found no difference in mortality with the addition of azithromycin to standard of care. In Brazil, Furtado et al. [23] found no difference in clinical status (odds ratio (OR) *=* 1.36, 95% confidence interval (CI) *=* 0.94, 1.97) or hospital length of stay (LOS) (26 vs 18 days; *P* = 0.064) was found among the treatment groups, however in Iran, Sekhavati et al. [24] found decreased hospital LOS (4.61 days vs 5.96 days; *P* = 0.02), and slightly higher SpO2 (93.95% vs92.40%; *P* = 0.030) was found in the azithromycin group. Notably, the standard of care in Brazil included hydroxychloroquine (HCQ) daily while in Iran included lopinavir/ritonavir (LPV/r) and HCQ.

### Antiparasitics

HCQ and chloroquine (CQ) were evaluated in four studies, including three RCTs. Among the RCTs, Abd-Elsalam et al. [25] and Cavalcanti et al. [27] found no benefit for HCQ on clinical status, need for MV, or mortality, while Borba et al. [26] found potential harm with higher risk of death (39% vs 15%; OR *=* 3.6, 95% CI = 1.2 to 10.6) among those treated with high-dose vs low-dose CQ. Both RCTs in Brazil found greater QTc prolongation with CQ and HCQ [26,27]. One retrospective cohort in China by Yu et al. [28] found lower mortality among HCQ treated patients (18.8% HCQ vs 47.4% non-HCQ; *P* < 0.001) but no difference in hospital LOS and with a serious risk of bias.

### Antivirals

Three RCTs and two retrospective cohort studies evaluated antivirals. Among RCTs, Abbaspour Kasgari et al. [29] evaluated sofosbuvir/daclatasvir with ribavirin vs LPV/r with or without ribavirin, Sadeghi et al. [32] evaluated sofosbuvir/daclatasvir, and Y Wang et al. [33] evaluated remdesivir; none of these studies found any benefit for the interventions studied on hospital LOS, mortality, or clinical status. In a retrospective cohort from Thailand, Prasithsirikul et al. [31] found no benefit for adjunctive favipiravir on hospital LOS (8 vs 10 days; *P* = 0.86) or time to viral clearance although this study had serious risk of bias due to multiple non-standardized therapeutics used in the comparator groups. Another retrospective cohort by Deng et al. [30] found greater viral clearance (75% vs 35%; *P* < 0.05) at seven days with use of umifenovir in addition to LPV/r vs LPV/r alone, although this study was seriously limited by small sample size (n = 16). Another retrospective cohort study in Iran by Vahedi et al. evaluated two different combinations of antibiotic plus antiviral plus other therapeutics found decreased hospital LOS (6.97 vs 9.93 days; *P* = 0.001) for azithromycin, prednisolone, naproxen, LPV/r vs meropenem, levofloxacin, vancomycin, HCQ and oseltamivir [34].

#### Convalescent plasma

Two RCTs by Agarwal et al. [35] and Li et al. [36] evaluated convalescent plasma; both found no benefit on clinical status or mortality, although Li et al. found possible benefit in clinical improvement in those with severe but non-critical disease with 91.3% vs 68.2% having clinical improvement at 28 days (HR = 2.15; 95% CI = 1.07 to 4.32).

#### Corticosteroids

Seven studies evaluated corticosteroids, including three RCTs and four retrospective cohort studies. Among RCTs, in Brazil, Jeronimo et al. [38] and Tomazini et al. [41] found no effect of steroids on mortality; however, Jeronimo et al. found decreased mortality in a sub-group analysis among patients over 60 years (46.6% vs 61.9%, *P* = 0.039) and Tomazini et al. found a higher number of ventilator-free days (6.6 vs 4.0 days; *P* = 0.04) among steroid-treated patients requiring mechanical ventilation (MV). In an RCT from Iran, Edalatifard et al. [37] found that among patients with elevated CRP and IL-6, steroids reduced hospital LOS (11.6 vs 17.6 days; *P* = 0.006), time to clinical improvement (11.8 vs 16.4 days; *P* = 0.011) and mortality (5.9% vs 42.9%; *P* < 0.001). In retrospective studies however, three studies in China found no difference in hospital LOS or mortality [39,42,43]. In Pakistan, Rana et al. [40] found improved Pao_2_:FiO2 (170 vs 118; *P* < 0.001) for dexamethasone vs methylprednisolone, however no other clinically relevant outcomes were evaluated.

#### Immunomodulators

Six studies evaluated immunomodulators including recombinant granulocyte colony-stimulating factor (rG-CSF), nasal interferon-kappa (IFN-k) with Trefoil factor 2 (TFF2), intravenous immunoglobulin (IVIG), IFN-a2b, IFN-b, and tocilizumab. One RCT by Cheng et al. [44] found no effect for rG-CSF on time to clinical improvement, however, lower 21-day mortality (secondary outcome) was seen in the treatment group (2% vs 10%), although was limited by small sample size. Among the two other RCTs, Gharebaghi et al. [46] found benefit for IVIG on in-hospital mortality (20% vs 48.3%; *P* = 0.022) and Rahmani et al. [48] found benefit for subcutaneous IFN-1b on time to clinical improvement (9 vs 11 days; *P* = 0.002) and hospital LOS (11 vs 13 days; *P* = 0.05), although no difference in 28-day mortality. A non-randomized trial by Fu et al. [45] found benefit for TFF-2 with IFN-k on hospital LOS (12 vs 15 days; *P* < 0.001), time to chest CT improvement (5 vs 8.5 days; *P* < 0.05) and time to viral clearance (6 vs 9.5 days; *P* < 0.05). Among retrospective studies, one cohort study by Zheng et al. found improvements in laboratory markers (WBC, CRP) with tocilizumab, but also longer hospital LOS (27.5 vs 16.4 days; *P* < 0.001) and higher mortality (9.8% vs 1.1%; *P* = 0.018), although patients who received tocilizumab were more likely to have severe/critical illness [49]. One case-control study by Hao et al. found no difference in viral clearance, need for MV or hospital LOS for IFN-a2b nasal spray [47].

#### Nutrients and minerals

One RCT by Abd-Elsalam et al. [50] evaluated the use of zinc with HCQ vs HCQ alone and found no difference in hospital LOS (13.5 vs 14 days; *P* = 0.553), clinical status (79.2% vs 77.9%, *P* = 0.969), or mortality (94.8% vs 94.7%; *P* = 0.986).

#### Respiratory tract agents

One RCT by de Alencar et al. evaluated the mucolytic agent N-acetylcysteine and found no difference in need for MV (20.6% vs 23.8%; *P* = 0.641), need for ICU admission (43% vs 47%; *P* = 0.557), or mortality (14% both groups; *P* = 0.940) [52]. Another RCT by Ansarin et al. assessed the mucolytic agent bromhexine and found decreased need for ICU admission (5.1% vs 28.2%; *P* = 0.006), need for MV (2.6% vs 23.1%; *P* = 0.007) and mortality (0% vs 12.8%; *P* = 0.027) [51].

#### Traditional Chinese medicine

TCM was evaluated in three RCTs and one retrospective cohort study, all from China. One RCT by Xiong et al. [56] evaluated Xuanfei Baidu, Wang et al. [54] evaluated Keguan Huo, and Xiao et al. [55] compared Huo Xiang Zhengqi plus Lianhua Qingwen vs Lianhua Qingwen alone vs standard of care alone (oseltamivir, arbidol, ribavirin). All RCTs included “Western medicine” in standard of care, although regimens were highly variable. Additionally, the primary outcomes varied substantially, and included time to symptom resolution, acute respiratory distress syndrome (ARDS) development, viral clearance, chest imaging improvement and progression to severe disease. Benefit was seen for Xuanfei Baidu with higher rate of symptom resolution at seven days (resolution of fever 90% vs 72.3%; *P* = 0.043) and for Keguan with decreased development of ARDS (4.2% vs 26.1%; *P* = 0.048) although this study had high concern for bias [54,56] The combination of Huo Xiang Zhengqi with Lianhua Qingwen had no benefit on progression to severe disease vs Lianhua Qingwen alone or standard of care (8.6% vs 1.6% vs 11.1%; *P* = 0.089). One retrospective study by Liu et al. found benefit for Jinhua Qinggan on time to viral clearance (7 vs10 days; *P* = 0.01) and time to chest imaging improvement (8 vs 10 days; *P* = 0.02) although the study had serious risk of bias [53]

### Community-acquired pneumonia and lower respiratory tract infections

There were 15 articles on CAP and LRTIs, including 11 RCTs and four observational studies (**Table 3**).

**Table 3:** Summary of findings on community-acquired pneumonia and lower respiratory tract infections (n = 15 studies)

Study ID	Country(s)	Design	Population	N	Intervention	Outcome	Key findings	RoB	
**Antibiotic**								
Abengowe, 1979 [57]	Nigeria	RCT	14-65 y with acute LRTI	126	Cotrimoxazole (TMP-SMX) 480-2400mg BID vs Tetracycline 500mg Q6H	Clinical improvement (composite of sputum characteristics, normal temperature, normal chest x-ray); side effects	Clinical improvement (in all three criteria) greater in treatment group (68.2% vs 36.5%, *P* < 0.01). x-ray changes resolved within 14 d more frequently in the treatment group (43 vs 23 patients, *P* < 0.001).	Some Concerns	
Chaudhary, 2009 [58]	India	RCT	≥18 y hospitalized with lower respiratory tract infection	240	IV Ceftazidime-Tobramycin 1g-120mg vs IV Ceftriaxone 1g	Clinical cure rate; chest radiograph improvement	Higher cure rate in 88.4% of the patients in Ceftazidime-Tobramycin FDC treated group as compared to 61.2% in cefatzidime alone treated group, with significant reduction in symptoms of dyspnoea, fever, cough, sputum, hemoptysis and chest pain in the patients	High	
Izadi, 2018 [59]	Iran	RCT	≥14 y hospitalized with CAP	150	PO Levofloxacin 750mg vs IV Ceftriaxone 1g BID + Azithromycin 250mg	Clinical Improvement (clinical signs and laboratory values); Hospital LOS	No difference in clinical improvement or hospital LOS (3.3 ± 0.7 vs 3.4 ± 0.6, *P* = 0.15) between levofloxacin monotherapy vs ceftriaxone + azithromycin.	Some Concerns	
Loh, 2005 [60]	Malaysia	Prospective cohort	>12 y with CAP	141	Addition of macrolide to a broad-spectrum antibiotic within 24 h of admission	In-hospital mortality	No difference in mortality with addition of macrolide (non-severe pneumonia, 6.5% vs 5.4%, *P* = 0.804; severe pneumonia, 17.6% vs 18.2%, *P* = 0.966). No difference in median hospital LOS (non-severe pneumonia, 5.5 vs 5 d, *P* = 0.954; severe pneumonia, 7 vs 6 d, *P* = 0.401).	Moderate	
Mendonça, 2004 [61]	Brazil	RCT	≥18 y hospitalized with mild to moderately severe pneumonia	51	IV/PO Gatifloxacin 400mg daily vs IV Ceftriaxone 1-2g daily (with or without macrolide)	Clinical cure rate (cure, failure, undetermined)	No difference in clinical cure rate between gatifloxacin vs ceftriaxone (92% and 88%).	Some Concerns	
Tieying, 2014 [62]	China	RCT	≥18 y with CAP and aspiration risk factors	77	IV Moxifloxacin 400mg daily vs IV Levofloxacin 500mg daily + Metronidazole 500mg BID	Clinical cure rate at 7-14 d	Clinical cure at 7 d after treatment were 76.7% for the moxifloxacin-treated patients, compared with 51.7% for the levofloxacin plus metronidazole-treated patients (χ^2^ *=* 4.002, *P* = 0.045). No difference in cure rate for moxifloxacin (83.3%) vs levofloxacin/metronidazole (71.8%) (*P* = 0.233) at end of treatment period.	High	
Wang, 2013 [63]	China	RCT	18-70 y with LRTI	272	IV Biapenem 300mg BID vs IV Meropenem 500mg Q8H	Clinical efficacy	No significant difference in clinical efficacy between biapenem and meropenem (94.7% vs 93.75%) for lower respiratory tract infection	Low	
Zhao, 2014 [64]	China	RCT	18-70 y with CAP	223	IV Levofloxacin 750mg for 5 d vs 500mg for 7-14 d	Clinical cure rate	There was no significant difference between the overall cure rate (56% vs 56.8%; difference = -0.8; 95% CI = -13.9 to 12.3) or efficacy rate (1.6%; 95% CI = -7.8 to 10.9) between levofloxacin 750mg for 5 d vs 500mg for 7-14d	Low	
Zhong, 2015 [65]	China, India, South Korea, Taiwan, and Vietnam	RCT	≥18 y with radiographically confirmed pneumonia	771	IV Ceftaroline 600mg BID vs IV Ceftriaxone 2g	Clinical cure rate	Clinical cure rate greater in ceftaroline 600mg q12h vs ceftriaxone 2g q24h (84% vs 74%; 95% CI = 2.8 to 17.1)	Some Concerns	
**Corticosteroid**								
Iqbal, 2020 [66]	Pakistan	Retrospective cohort	≥18 y with CAP	508	IV Hydrocortisone 100mg Q8H then PO Prednisolone vs no steroids	In-hospital mortality, hospital LOS	No effect of steroids on in-hospital mortality (aOR:0.85, 95% CI: 0.39-1.88). IV steroid group had longer hospital LOS (IRR = 1.51, 95% CI = 1.37-1.66).	Moderate	
Nafae, 2013 [67]	Egypt	RCT	≥18 y with CAP	80	IV Hydrocortisone 200mg + infusion vs placebo	Pao_2_/FiO2 ratio	Improvement in Pao_2_:FiO2 ratio (365.5 ± 61.4 vs 321.5 ± 101.9), inflammatory markers (WBC, CRP, ESR), reduced hospital LOS (9.27 ± 2.4 vs 16.5 ± 2.24; *P* < 0.05) and deaths (6.7% vs 31.6%; *P* < 0.05) in adjuvant hydrocortisone vs placebo group.	Some Concerns	
**Guidelines**								
Annisa, 2014 [68]	Malaysia	Retrospective cohort	≥18 y with CAP	323	National guidelines on antibiotic use	Hospital LOS; clinical Improvement (clinical signs and laboratory values)	No difference in hospital LOS (4.72 vs 4.9 d, *P* = 0.457) or most clinical signs measured however, decreased time to resolution of tachycardia and leukocytosis in guideline-adherent vs non-adherent group (1.77 vs 2.45 d; *P* = 0.041) and (5.51 vs 1.16; *P* = 0.040).	Serious	
Silveira, 2012 [69]	Brazil	Retrospective cohort	≥18 y hospitalized with CAP	112	Adherence to Brazilian Thoracic Association guidelines	30-d mortality	No significant difference in 30-d mortality in guideline-concordant patients except for those with CRB-65 score 1-2 (*P* = 0.01). Non-significant difference in hospital LOS in patients in whom admission and treatment criteria were in accordance with guidelines (12d vs 16d; *P* = 0.066). Multivariable regression showed lower risk of death in guideline-adherent group (RR = 0.85, 95% CI = 0.76 to 0.96)	Moderate	
**Traditional Chinese Medicine**								
Song, 2019 [70]	China	RCT	18-75 y old with severe CAP	710	XueBiJing 100mL BID vs placebo	Clinical Improvement (pneumonia severity index); 28-d mortality	Improvement in the pneumonia severity index risk (60.78% XueBiJing vs 46.33% placebo) (between-group difference, 14.4% (95% CI = 6.9‚ 21.8%); *P* < 0.001). Lower 28-d mortality rate (15.87% XueBiJing vs 24.63%; *P* = 0.006)	Some Concerns	
**Nutrients and Minerals**								
Sharafi, 2016 [71]	Iran	RCT	≥50 y wth CAP	89	Zinc sulphate 110mg BID vs placebo	Hospital length-of-stay	No significant difference in LOS (*P* = 0.18), normalization of RR (*P* = 0.55) and SpO2 (*P* = 0.26) between zinc vs placebo group (*P* = 0.18).	Some Concerns	

BID – twice daily, CI – confidence intervals, CT – computed tomography, ICU – intensive care unit, IV – intravenous, LOS – length of stay, mg – milligram, N – number of participants, PO – per oral, RoB – risk of bias, RR – respiratory rate, SpO2 – oxygen saturation, IRR – incidence rate ratio

#### Antibiotics

Nine of the 15 studies evaluated antibiotic regimens. Four of the eight RCTs evaluating antibiotics found no difference in clinical efficacy when comparing the following antibiotic regimens: gatifloxacin vs ceftriaxone (92% and 88% cure rate; *P*-value not reported), biapenem vs meropenem (94.7% vs 93.75% clinical efficacy; *P*-value not reported), levofloxacin five day vs seven day course (cure rate 56% vs 56.8%; difference = -0.8; 95% CI = -13.9 to 12.3) and levofloxacin vs ceftriaxone with azithromycin (hospital LOS 3.3 vs 3.4; *P* = 0.15) [59,61,63,64]. Abengowe et al. [57] found higher cure rates (68.2% vs 36.5%; *P* < 0.01) with TMP-SMX over tetracycline in Nigeria, and Chaudhary et al. [58] found benefit for ceftazidime-tobramycin over ceftriaxone in India (88.4% vs 61.2%; *P*-value not reported), although with high risk of bias. Tieying et al. [62] found that clinical cure at seven days was greater for moxifloxacin over levofloxacin/metronidazole (76.7% vs 51.7%; *P* = 0.045) among patients with CAP with aspiration risk factors at seven days, although no difference at the end of the treatment period at 14 days (83.3% vs 71.8%; *P* = 0.233). In a five-country non-inferiority study in Asia, Zhong et al. [65] found higher clinical cure rate for ceftaroline vs ceftriaxone (84% vs 74%, 95% CI *=* 2.8 to 17.1). Two retrospective cohort studies found no difference in hospital LOS or mortality between national antibiotic guideline-concordant or guideline-discordant groups, although in Brazil, Silveira et al. found a trend toward shorter LOS (12 vs 16 days; *P* = 0.066) in guideline-concordant patients and in multivariable analysis, a lower risk of 30-day mortality (RR = 0.85, 95% CI = 0.76 to 0.96) in guideline-concordant patients [68,69]. One prospective cohort study in Malaysia by Loh et al. [60] found no benefit to addition of a macrolide to broad-spectrum antibiotics on hospital mortality (17.6% vs 18.2%; *P* = 0.966) or LOS (7.0 vs 6.0 days, *P* = 0.401).

#### Other interventions

One RCT by Song et al. evaluated the TCM treatment XueBiJing, hypothesized to have anti-inflammatory and immunomodulatory effects, and found improvements in pneumonia severity index (60.8% vs 46.3%;  *P* < 0.001and lower 28-day mortality (15.87% XueBiJing vs 24.63%; *P* = 0.006) [70]. One RCT by Sharafi et al. evaluated the use of adjunctive zinc in older patients >50 years with CAP and found no benefit for use of zinc on hospital LOS [71]. Nafae et al. evaluating corticosteroids in an RCT in Egypt which showed reduced mortality (6.7% vs 31.6%; *P* < 0.05), and reduced hospital LOS (9.3 vs 16.5 days; *P* < 0.05) among patients treated with adjuvant hydrocortisone [67]. However, in a retrospective cohort study in Pakistan, Iqbal et al. found no effect of steroids on hospital mortality (absolute odds ratio (aOR) *=* 0.85, 95% CI *=* 0.39 to 1.88) and found the steroid group had longer hospital LOS (incidence rate ratio (IRR) = 1.51, 95% CI *=* 1.37 to 1.66) although was limited by lack of data on timing of steroid administration [66].

### Influenza (including H1N1 and H7N9)

There were seven studies on influenza: six evaluated neuraminidase inhibitors (NAIs) and one evaluated corticosteroids (**Table 4**). Among the six studies on NAIs, two were RCTs and four were retrospective cohort studies; five were conducted in China. Among RCTs, Li et al. [75] found shorter duration until symptom resolution with oseltamivir vs placebo; however, the magnitude of effect varied with one study showing a reduction in duration of symptoms of less than five hours (91.6 vs 95 hours; *P* = 0.0466) while Lin et al. [76] found a reduction of 64 hours (110 vs 174 hours; *P* = 0.0479). Three of the retrospective cohort studies evaluating NAIs found benefit for oseltamivir. Chen et al. [73] found a decreased need for MV (OR = 0.325, 95% CI = 0.123 to 0.858) and mortality (OR = 0.416, 95% CI = 0.184 to 0.944) with early initiation (within two days of symptom onset) of oseltamivir in Influenza B, and also similarly found decreased need for MV (OR = 0.511, 95% CI = 0.312 to 0.835) and mortality (OR = 0.533, 95% CI = 0.210 to 0.807) with early oseltamivir treatment in Influenza A [72]. Lenzi et al. [74] also found decreased mortality (OR = 0.031, 95% CI = 0.015 to 0.065) with oseltamivir treatment. In a retrospective cohort study, Zhang et al. evaluated peramivir but found no benefit for the addition of peramivir to oseltamivir on development of time to viral clearance (7.0 vs 6.5 days; *P* = 0.67), development of ARDS (77.8% vs 63.9%; *P* = 0.30) or mortality (43.6% vs 25.6%; *P* = 0.11) [77]. Cao et al. [78] performed a propensity score-matched case-control study and found that high-dose adjuvant steroid use was associated with higher mortality at 30 days (adjusted hazard ratio (aHR) = 3.05, 95% CI = 1.28 to 7.25) in patients with H7N9 influenza while no association was seen with low or moderate doses.

**Table 4:** Summary of findings on influenza (including H1N1 and H7N9 (n = 7 studies))

Study ID	Country(s)	Design	Population	N	Intervention	Outcome	Key findings	RoB
**Antivirals**								
Chen, 2020 [72]	China	Retrospective cohort	>14 y hospitalized with influenza B	386	Any NAI administered within 2 d after symptom onset	Invasive and non-invasive mechanical ventilation, admittance to the ICU, and 30-d mortality	Early NAI treatment was associated with the decreased risks of invasive ventilation (OR = 0.325,95% CI = 0.123 to 0.858; *P* = 0.023), admittance to ICU (OR = 0.425, 95% CI = 0.204 to 0.882; *P* = 0.022), and 30-d mortality (OR = 0.416, 95% CI = 0.184 to 0.944, *P* = 0.036).	Moderate
Chen, 2020 [73]	China	Retrospective cohort	>14 y hospitalized with Influenza A	693	Any NAI administered within 2 d after symptom onset.	Invasive ventilation,14-d and 30-d mortality	Early NAI therapy associated with decreased risk for invasive ventilation (OR = 0.511, 95% CI = 0.312 to 0.835) and 30-d mortality (OR = 0.533, 95% CI = 0.210 to 0.807).	Serious
Lenzi, 2012 [74]	Brazil	Retrospective cohort	Any age hospitalized with H1N1 influenza	1917	Treatment with Oseltamivir	Clinical cure; time to death	Decreased mortality with oseltamivir in regression (OR = 0.031, 95% CI = 0.015 to 0.065). Average time-to-death in untreated patients was 8.6 d vs 13.8 d in those who received oseltamivir; 32.3% increased risk of death for each additional day to oseltamivir initiation after symptom onset.	Moderate
Li, 2003 [75]	China	RCT	18-65 y with symptoms of influenza	451	PO Oseltamivir 75 mg BID vs placebo	Duration of symptoms; symptom severity score	Shorter time to symptom resolution in oseltamivir group vs placebo (*P* = 0.0466) with duration of illness 91.6 h (95% CI = 80.2 to 101.3) vs 95 h (95% CI = 84.5 to 105.3) and lower symptom severity (*P* = 0.0196).	Some Concerns
Lin, 2006 [76]	China	RCT	Adults with chronic respiratory or cardiac disease with influenza six within 48 h of symptom onset	56	PO Oseltamivir 75 mg BID vs placebo	Duration of symptoms; symptom severity score	Oseltamivir reduced duration of symptoms by 64 h or 36.8% (110 vs 174 h; *P* = 0.0479) and severity by 43.1%. Reduced time to baseline health status by 5 d (*P* = 0.0011), incidence of complications (11% vs 45%, *P* = 0.0053) and antibiotic use (37% vs 69% *P* = 0.0167).	High
Zhang, 2016 [77]	China	Retrospective cohort	≥18 y with H7N9 Influenza A (H7N9) virus	82	PO Oseltamivir + IV Peramivir vs PO Oseltamivir alone	Time to viral clearance; mortality; development of ARDS	No difference time to viral clearance (7.00 vs 6.50 d, *P* = 0.67), development of ARDS (77.78% vs 63.89%, *P* = 0.30) or mortality (43.6% vs 25.6%, *P* = 0.11) oseltamivir-peramivir vs oseltamivir monotherapy.	Moderate
**Corticosteroids**								
Cao, 2016 [78]	China	Case-control	>14 y hospitalized with severe H7N9	288	At least one dose of corticosteroids equivalent to 25mg methyprednisolone	30-d and 60-d mortality	Increased 30-d (aHR = 3.05; 95%CI = 1.28 to 7.25; *P* = 0.012) and 60-d mortality in high-dose steroid (aHR = 4.05; 95% CI = 1.82 to 9.02); *P* = 0.001) but no effect with low or moderate dose. Corticosteroid cases had higher 60-d mortality vs no steroids (aHR = 1.98; 95% CI = 1.03 to 3.79; *P* = 0.04)	Serious

aHR – adjusted hazard ratio, ARDS – acute respiratory distress syndrome, BID – twice daily, CI – confidence interval, HR – heart rate, ICU – intensive care unit, IV – intravenous, LOS – length of stay, NAI – neuraminidase inhibitor, N – number of participants, OR – odds ratio, PO – per oral, RoB – risk of bias, RR – respiratory rate, ICU – intensive care unit

#### Severe acute respiratory infection

Only one study used the SARI definition as inclusion criteria, a double-blind RCT in Mexico by Gamiño-Arroyo et al. [79] which found no difference in hospital LOS (6.5 vs 7.0 days; *P* = 0.56) or viral clearance at day 3 (21.5% vs 21.3%; *P* = 0.98) in patients treated with the antiparasitic nitazoxanide (shown to have in vitro antiviral activity) (Table 5).

**Table 5:** Summary of findings on severe acute respiratory infections and severe acute respiratory syndrome (n = 2 studies)

Study ID	Country(s)	Study Design	Population	Disease	N	Intervention	Outcome	Key findings	RoB
**SARI**									
Gamiño-Arroyo, 2019 [79]	Mexico	RCT	Patients over 1 y requiring hospitalization because of an acute influenza like illness	SARI	257	Nitazoxanide (NTZ) 600mg BID x 5 d vs placebo	Hospital LOS	No significant difference in hospital LOS between NTZ group 6.5 (IQR = 4.0-9.0) days vs 7.0 (IQR = 4.0-9.0) days in the placebo group (*P* = 0.56). No difference in viral clearance (21.5% vs 21.3%; *P* = 0.98)	Low
**SARS**									
Chen, 2006 [80]	China	Retrospective cohort	Patients with laboratory-confirmed SARS admitted to hospital	SARS	401	Corticosteroids - “aggressive treatment” for critical cases or rescue regimen (sudden increase in dose after 3 weeks from onset and 3 d before death) for worsened cases	Mortality; hospital LOS	No difference in mortality or hospital LOS for steroid vs no steroids for overall study population. In critical cases, lower mortality (OR = 0.083, 95% CI = 0.007-0.956) and shorter hospital LOS (OR = 1.743, 95% = 1.025-2.964).	Moderate

BID – twice daily, IQR – interquartile range, CI – confidence interval, LOS – length of stay, NAI – neuraminidase inhibitor, N – number of participants, OR – odds ratio, RCT – randomized controlled trial, RoB – risk of bias, SARI – severe acute respiratory infection, SARS – severe acute respiratory syndrome

#### Severe acute respiratory syndrome

One retrospective cohort study in China by Chen et al. [80] found no difference in mortality or hospital LOS between patients with SARS who received corticosteroids and those who did not; however, among patients with critical illness, there was lower mortality (OR *=* 0.083, 95% CI *=* 0.007 to 0.956) and shorter hospital LOS (OR *=* 1.743, 95% CI *=* 1.025 to 2.964) with corticosteroid use although the exact steroid dosages and timing of administration were not described (**Table 5**).

## DISCUSSION

This systematic review broadly evaluated the impact of acute and emergency care interventions on clinical outcomes of adolescents and adults meeting the SARI case definition in LMICs. We found a critical lack of evidence exists for best practices in the management of undifferentiated SARIs across resource-limited settings, despite the overall low- to moderate-quality evidence for the benefit of early administration of certain pharmacologic interventions (eg, antivirals, antibiotics, immunomodulators) on the outcomes of patients with specific aetiologies of SARI (eg, influenza, COVID-19). None of the included studies evaluated non-pharmacologic interventions or were conducted in LICs.

One of this review’s strengths was the use of the SARI definition as an inclusion criterion, mimicking the real-world nature of evaluating patients with initially undifferentiated respiratory infections and avoiding reliance on laboratory diagnostics or suspicion of a specific pathogen as inclusion criteria. Multiple respiratory viruses such as influenza, SARS, and SARS-CoV-2, as well as (presumably) bacterial causes of pneumonia (few studies included information on confirmatory microbiologic diagnoses for studies on pneumonia) were included by these criteria. This also ensured that all relevant studies on SARIs would be included in the review, even if the SARI term itself was not used (eg, if the study was published before the SARI definition was created in 2011). The greatest variety of interventions were found among COVID-19 studies, while interventions for other SARIs were largely limited to antibiotics and antivirals. Not surprisingly, this review was dominated by the recent surge of publications related to COVID-19, as noted in multiple other fields, with only 21 of the 58 included articles (36%) published prior to 2020 [81,82].

All but one study required preliminary or confirmatory diagnosis of the likely aetiologic pathogen(s) causing SARI, usually via laboratory testing and/or radiologic imaging. While this helped ensure homogeneity of the study population relevant for targeted therapeutics such as antivirals or antibiotics, aetiologies of SARIs are often unknown to clinicians during the early period of care due to limited diagnostic capacities in LMICs. This review has highlighted the need for research on SARIs in LMICs using clinically based inclusion criteria, and increased focus on undifferentiated patient populations, prior to or without etiologic diagnostic test result requirements. Three major disease processes dominated the review – COVID-19, influenza, and CAP. Notably, all the interventions studied were pharmacologic in nature.

Studies on viral respiratory diseases were largely focused on the use of antivirals, while those on CAP focused on comparisons of antibiotic regimens. Most studies on CAP showed a non-inferiority of the studied antibiotics compared to control groups and mixed evidence on the utility of steroids in CAP. The most consistent, highest-quality evidence was on the benefit of NAIs for influenza treatment. NAIs were found to reduce symptom duration, clinical severity, and decreased patients’ risk of needing MV and risk of death. Despite the relative strength of these findings, substantial debate exists regarding the effectiveness and safety of NAIs for influenza and the appropriateness of stockpiling these drugs for use in influenza pandemics [83,84]. Much of this debate has been based on several large systematic reviews and meta-analyses (including one Cochrane collaboration report) which included data from unpublished manufacturer-funded trials [85-87]. These reviews found that, despite a significant but short (approximately half a day) reduction in symptom duration, there remains a lack of credible data on the effect of NAIs on the outcomes of greatest clinical interest, such as reducing severe complications of influenza, risk of hospitalization, or death [85-87]. Additionally, given that five of the six included studies on NAIs in this review were conducted in a single upper-middle income country (China), further research on the utility of NAIs in other LMICs, and focusing on outcomes of hospitalization and death, are greatly needed.

Among studies on COVID-19, little to no benefit was seen for most of the proposed therapeutics. Overall, the studies show a lack of benefit for azithromycin, HCQ and CQ, and convalescent plasma; mixed results were found with certain antivirals, corticosteroids and immunomodulators, with some studies noting differential effects among certain sub-groups (eg, corticosteroids for severe illness). However, the lack of standardization of a standard-of-care for control groups across countries or within the same country at different time points of the pandemic makes the extrapolation of these findings to other populations or contexts extremely difficult. Internationally standardized and evidence-based guidelines for the care of COVID-19 patients enrolled in research studies appropriate for LMIC settings (where access to supportive care such as supplemental oxygen may not be guaranteed) are needed. Importantly, given the volume of publications since the conclusion of this review’s search, an updated review of the literature on acute and emergency care interventions for COVID-19 in LMICs is warranted and is planned by this study team. Additionally, as this study was conducted before the roll-out of COVID-19 vaccines, the magnitude of effect of the interventions studied may differ among vaccinated individuals.

Five of the included studies evaluated the impact of TCM on SARIs, all conducted in China. All but one study found benefit of TCM on outcomes; however, the primary outcomes were highly variable, with most focusing on metrics such as symptom resolution or chest imaging improvements and only one including mortality as an outcome. Given the cultural context of TCM and limited use outside of East Asia, as well as the concomitant use of TCM alongside “Western medicine” in these studies, it is unclear how TCM interventions would be applied practically in other settings [88]

Unfortunately, no studies evaluated other intervention types such as oxygen administration strategies, ventilation strategies, or other supportive care strategies. While it is possible that this search strategy missed some studies, we believe this is not the case, as numerous non-pharmacologic interventions identified in this group’s concurrent paediatric-focused review (to be published separately) used the same search strategy; in the paediatric-focused review, more than 20% of the included articles evaluated respiratory support or physiotherapy interventions. Additionally, no additional relevant studies were found during cross-checking of reference lists of the articles. Lastly, we know that there is a lack of studies evaluating non-pharmacologic interventions for SARI in LMICs.

This study’s findings are mostly consistent with existing guidelines on the management of patients with respiratory infections in LMICs, which have focused primarily on pharmacologic (antibiotic and antiviral) treatments[89,90]. Resources such as WHO’s Clinical Care for SARI toolkit have been a great asset in the rapidly changing context of COVID-19 with inclusion of a living guideline on COVID-19 therapeutics and specific ventilation strategies for SARI, although guidance remains largely extrapolated from research conducted in high-resource settings rather than LMICs [6]. Additionally, recent guidelines created by and for those working primarily in resource-limited emergency care settings have increased emphasis on the care of undifferentiated severe respiratory conditions and the provision of context-appropriate supportive care, with tailored recommendations corresponding to different levels of resource availability [91,92]. While these developments are encouraging, incorporation of high-quality evidence obtained from LMICs into guidelines for best practices in the early care of patients with SARIs is urgently needed. One outcome of the COVID-19 pandemic may be the increased global attention toward respiratory support and non-pharmacologic interventions on altering the course of SARIs. This recognition of its importance may hopefully translate to increased resources and infrastructure needed to support emergency and critical resources in LMICs, such as enhanced systems of oxygen delivery.

This review has several important limitations. Early in the development of this review, the study team faced challenges in defining the inclusion criteria in several respects. First, as the SARI definition includes “requires hospitalization,” studies in populations with few hospital-level facilities available may have been excluded, skewing study findings towards places with greater health care resources. Another challenge was in determining what interventions constituted “emergency care”. Given the scope of emergency care may not be clearly defined in many LMICs, the broad definition of “emergent and early interventions” led to a wide scope of interventions being included, such as those that may not clearly represent emergency care in which medication started within the first 24 hours of care. Additionally, few studies explicitly reported on the exact timing of interventions (eg, within the first few hours vs anytime during the first day of admission) making assessment of what counted as emergency care difficult. The authors opted to err on the side of caution (ie, increased inclusivity) where uncertainty existed and therefore some interventions may not strictly be considered emergency care in all contexts. As timing of interventions may greatly impact the trajectory of disease progression, future research on early and emergency care of patients should report these key metrics in publications. Additionally, some studies’ primary outcomes focused on metrics (such as non-inferiority) which may be less relevant to emergency care clinicians. Lastly, the broad nature of the conditions studied using the SARI definition, interventions, and outcomes made meta-analysis including pooling effect estimates unfeasible, as mentioned previously.

Since all LMICs were included in this review, the applicability of certain interventions, such as convalescent plasma (which require special blood bank services) or highly costly immunomodulators, to lower resourced settings are likely very limited. Given the heterogeneity in resources available in countries classified as upper-middle income vs low- or lower-middle incomes, reviews focused broadly on LMICs should consider classifying findings according to resource availability. A glaring gap identified in this review was the lack of any included studies conducted in LICs. The reason for the lack of data from LICs is unclear, but may be due to lower research capacity or funding for interventional trials, lower SARI surveillance capacity or a lower burden of epidemic-potential SARIs. It is also possible that adult-focused research has been of lesser priority in LICs, given more than 12% of articles in the concurrent paediatric focused analysis were conducted in LICs It is crucial that patients in LICs have equal opportunities for participation in research trials for SARI therapeutics and interventions, especially in the context of COVID-19. Lastly, studies on recent SARI outbreaks that primarily affected high-income countries (eg, MERS and SARS) were not included.

## CONCLUSIONS

Despite a significant increase in research on the treatment of SARI since the start of the COVID-19 pandemic, few interventions for SARI in LMICs had high quality evidence for benefit on clinical outcomes. NAIs for influenza had the strongest evidence for decreasing symptom duration and disease severity although research in more diverse populations is needed. Further research evaluating non-pharmacologic interventions, immunomodulators, corticosteroids, and including emergency care-relevant metrics for patients with SARIs in low-resource settings in LMICs are greatly needed.

## Additional material:


Online Supplementary Document
