# Supplementary material for: A systematic review of acute and emergency care interventions for adolescents and adults with severe acute respiratory infections including COVID-19 in low- and middle-income countries
Source: J Glob Health. 2022 Nov 8;12:05039. doi: 10.7189/jogh.12.05039 (PMC9639745; doi:10.7189/jogh.12.05039)
Supplement: Online Supplementary Document [file jogh-12-05039-s001.pdf]

ONLINE SUPPLEMENTARY DOCUMENT

Title: A Systematic Review of Acute and Emergency Care Interventions for Adolescents and Adults with Severe Acute Respiratory Infections Including COVID-19 in Low- and Middle-Income Countries

Authors:
Stephanie Chow Garbern MD MPH
Pryanka Relan MD MPH
Gerard M O'Reilly MBBS FACEM MPH MBiostat AStat PhD
Corey B Bills MD MPH
Megan Schultz MD MA
Indi Trehan MD MPH DTM&H
Sean M Kivlehan MD MPH
Torben K Becker MD PhD

Supplemental Figure S1. Consensus judgements using Cochrane risk of bias for randomized trials version 2 (RoB 2) for each included study. Each risk of bias item are assigned a color-coded ranking; green color represents low risk of bias, yellow some concerns, and red high risk of bias.

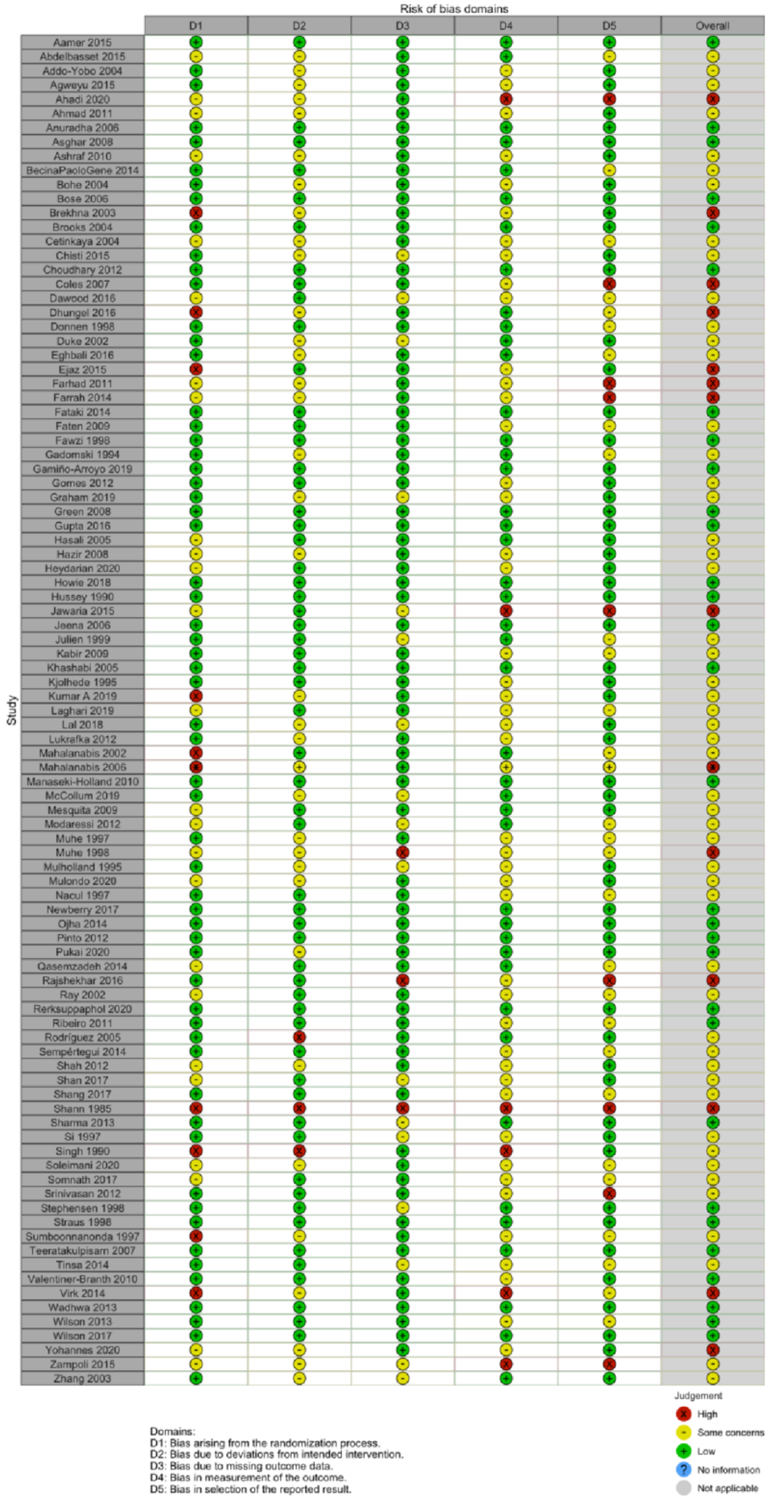

Supplemental Figure S2. Consensus judgements using Cochrane ROBINS-I for each included nonrandomized study. Each risk of bias item are assigned a color-coded ranking; green color represents low risk of bias, yellow some concerns, and red high risk of bias.

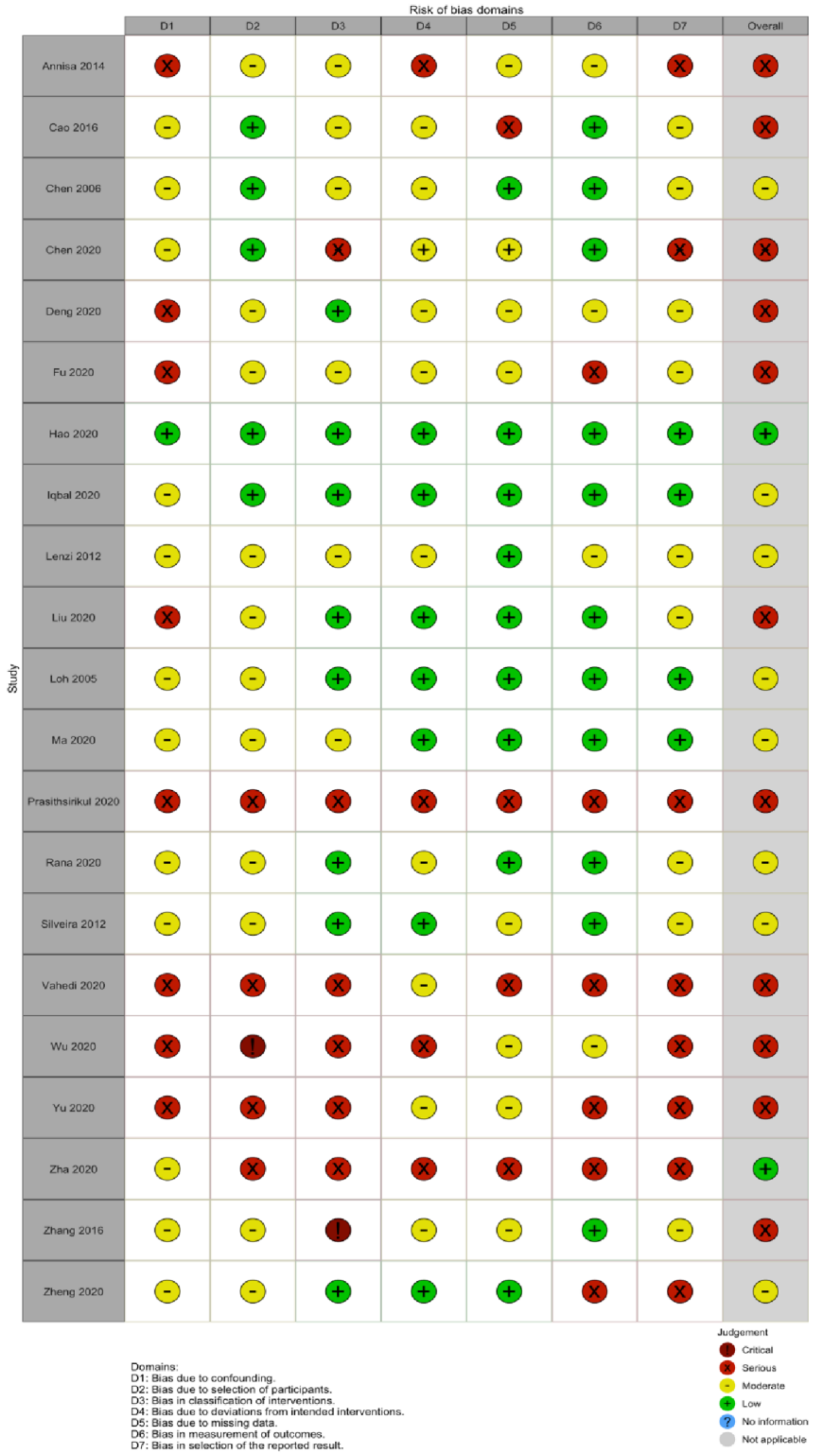

Supplemental Figure S3. Example of six-point Clinical Status ordinal scale used in COVID-19 studies.

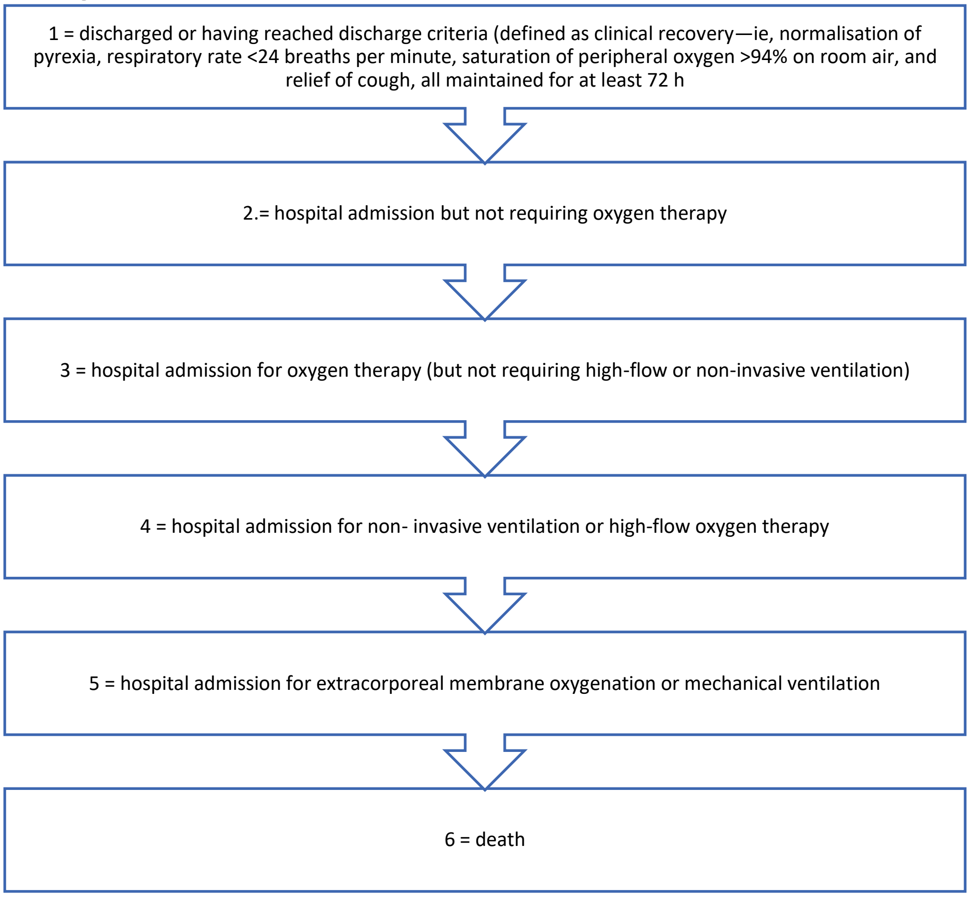

Appendix S1

Systematic Review Protocol

Title: Impact of Emergency Care Interventions on Severe Acute Respiratory Infection Outcomes in Low- and Middle-Income Countries: A Systematic Review

Authors:
Stephanie C. Garbern MD MPH[1], Corey B. Bills MD MPH[2], Gerard O'Reilly MBBS FACEM MPH MBiostat AStat PhD[3,4]; Pryanka Relan MD MPH[5], Megan L. Schultz MD MA[6], Sean M. Kivlehan MD MPH[7], Indi Trehan MD MPH DTM&H[7,8], Torben K Becker MD PhD[9]

1. Department of Emergency Medicine, Warren Alpert Medical School of Brown University, Providence, RI, USA

2. Department of Emergency Medicine, University of Colorado School of Medicine, Aurora, CO, USA
3. Emergency and Trauma Centre, The Alfred, Melbourne, Australia
4. School of Public Health and Preventive Medicine, Monash University, Melbourne, Australia
5. Department of Emergency Medicine, Emory Healthcare Network, Atlanta, GA, USA
6. Department of Pediatrics, Medical College of Wisconsin, Milwaukee WI
7. Department of Emergency Medicine, Brigham and Women's Hospital, Boston, MA, and Harvard Humanitarian Initiative, Cambridge, MA.
8. Lao Friends Hospital for Children, Luang Prabang, Lao, PDR
9. Departments of Pediatrics and Global Health, University of Washington, Seattle, WA, USA
10. Department of Emergency Medicine, University of Florida, Gainesville, FL.

Corresponding Author:
Stephanie C. Garbern, MD MPH
Department of Emergency Medicine
Alpert Medical School of Brown University
55 Claverick Street, 2nd Floor Providence,
RI 02906
sgarbern@brown.edu

Guarantor: Dr. Torben Becker

Registration: PROSPERO

Logistical Support: Global Emergency Medicine Literature Review (GEMLR) Group

Financial Support: None

Conflicts of Interest: None

INTRODUCTION:
Rationale:

Acute respiratory infections remain a leading cause of morbidity and mortality in low- and middle-income countries (LMICs). Certain viral etiologies of Severe Acute Respiratory Infections (SARI) pose an additional pandemic potential, such as pandemic influenza A (H1N1/09), Middle East respiratory syndrome coronavirus (MERS-CoV) and severe acute respiratory syndrome coronavirus 2 (SARS-CoV2), threatening patients worldwide, with those in LMICs often the most vulnerable. Despite steady advances in characterizing the etiologies, incidence, and factors contributing to severe acute respiratory infections (SARI), knowledge gaps persist in identifying which emergency care interventions, are most effective in improving patient outcomes in LMICs. Filling these gaps is critical to ensuring that limited available resources can be optimally targeted towards feasible, effective interventions.

Critical Review of Relevant Literature:

Review Question:

What is the impact of emergency care interventions on improving the health outcomes of patients with severe acute respiratory infections in LMICs?

Condition or domain being studied:
Severe acute respiratory infections (SARI), as defined by the World Health Organization, are acute respiratory infections with a history of fever or measured fever of ≥ 38 C°; and cough; with onset within the last 10 days; and require hospitalization.

METHODS:
Eligibility Criteria:

Studies from all years in English or Spanish evaluating the impact of emergency care interventions on SARI health outcomes will be included. See Table 1.

Table 1. PICO style presentation of systematic review

**Criterion**	**Included**	**Excluded**
**Population**	Patients with Severe Acute Respiratory Infections in LMICs<br>• Pediatric (0-19)<br>• Adult (>19 years)<br><br>Severe Acute Respiratory Infection definition (WHO):<br><br>An acute respiratory infection with:<br>• history of fever or measured fever of ≥ 38 C°;<br>• and cough;<br>• with onset within the last 10 days;	Patients in High Income Countries (World Bank Classification)<br><br>Neonates (<28 days, prematurity corrected)<br><br>Patients with chronic respiratory infections without acute decompensation<br><br>Patients with mild respiratory infections not requiring hospitalization
	• and requires hospitalization (supplemental oxygen or interventions to prevent decompensation or death)<br><br>(This would include sepsis, acute respiratory distress syndrome (ARDS), Acute Respiratory Failure **due to a Respiratory Infection**)	Patients with sepsis or ARDS NOT due to a respiratory infection (non-respiratory or “undifferentiated” source of infection/sepsis)

**Intervention**	Emergency Care Interventions<br>• Medications (antibiotics, antivirals, steroids, micronutrients, inhaled medications)<br>• Respiratory support (oxygen, noninvasive and mechanical ventilation, continuous positive airway pressure (CPAP), high flow oxygen)<br>• Other supportive care (proning, etc.)<br>• Triage Tools<br>• Monitoring (pulse oximetry, etc.)<br>• Educational interventions<br><br>Emergency Care:<br>• Interventions that provide or facilitate early and time-sensitive care of acutely ill patients, whether outside or inside a healthcare facility (including early critical care)	• Diagnostic Tools<br>• Chronic disease management interventions<br>• Preventative care<br>• Severity Scores<br>• Prognostic Scores<br>• Vaccines
**Control**	Required	No Control
**Outcome**	Clinical outcomes (measurable changes in health, function or quality of life)<br>• Mortality<br>• Disease progression<br>• Need for ICU level of care<br>• Need for mechanical ventilation<br>• Hospital length-of-stay<br>• Adverse events<br>• Other relevant emergency care clinical outcomes	• Knowledge / educationbased outcomes<br>• Cost-effectiveness<br>• Health System outcomes<br>• Public Health outcomes<br>• Disease Prevention
**Study Type**	• Randomized control trials<br>• Observational studies with a control group	• Case reports<br>• Case series<br>• Opinion papers<br>• Descriptive papers
	• Observation studies with a pre/post design	• Abstract Only
**Language**	English, Spanish	
**Year of Publication**	All	

We will define low- and middle-income countries using the World Bank country classification from

2019-2020. World Bank country classification based on income is updated July 1 of every year. Since 2010, 15 countries have reclassified as 'High Income' countries, meaning that publications utilizing data obtained after country reclassification will be excluded. By year, the following countries have been reclassified as high income. 2019: Mauritius, Nauru, Romania. 2018: Argentina, Croatia, and Panama. 2017: Palau. 2016: British Virgin Islands, Gibraltar, Nauru. 2013: Antigua and Barbuda, Chile, Latvia, Lithuania. 2012: St. Kitts and Nevis. 2011: Curacao and St. Martin.

Exclusion criteria:

1. Study was not undertaken in an LMIC
2. Study primarily in neonates (age < 28 days)
3. Study in patients without SARI (i.e. those with mild illness, chronic respiratory diseases, respiratory failure not primarily due to respiratory infection, etc.)
4. Not an emergency care intervention (i.e. chronic disease management or preventative care e.g. vaccines); diagnostic tools; severity/prognostic scores)
5. Outcome is non-clinical (i.e. cost-effectiveness, health systems management/process outcomes, knowledge-based/education only outcomes; disease prevention outcomes; population-level/public health outcomes)
6. No control / comparison group (i.e. case reports, descriptive studies without control; opinion papers)
7. Article not in English or Spanish

1. 8. Abstract only

Inclusion Criteria:
Pediatric (0-19 years) and Adult (>19 years) Patients with Severe Acute Respiratory Infections in low- and middle-income countries.

We will define low- and middle-income countries using the World Bank country classification from 2019-2020.

Exclusion Criteria:
Patients in High Income Countries (World Bank Classification)
Neonates (<30 days, prematurity corrected)
Patients with chronic respiratory infections without acute decompensation
Patients with mild respiratory infections not requiring hospitalization
Patients with sepsis or acute respiratory distress syndrome (ARDS) not due to a respiratory infection

Information Sources:

Literature sources will include PubMed, Embase, OVID: Global Health (CABI), LILAC, SciELO, Web of Science, and the Global Index Medicus (WHO). Grey literature will be included as well via advanced google searches targeting organizations known to publish global emergency care literature including but not limited to the World Health Organization, Doctors Without Borders, International Committee of the Red Cross, and the International Rescue Committee. The references of included articles and main review articles will be reviewed in search of additional studies.

Search Strategy:

Search terms were developed via literature review, use of current Cochrane/WHO guidelines, and expert opinion. Search terms are available by request and will be made available in a published appendix.

Comparator/Control:
The control will be defined as placebo or routine care as defined by the author(s) of the article. The comparator will be defined as any emergency care intervention provided for the management of patients with SARI.

Context:
Studies of interventions that provide or facilitate the early care of patients with severe acute respiratory infections, whether outside or inside a health-care facility. Research conducted with patient populations living in low- and middle-income countries only will be included.

Data Extraction (Selection and Coding):
Covidence (Veritas Health Innovation, Melbourne, Australia), a web-based systematic review software platform, will be used to manage records and record decisions regarding study selection for inclusion in the systematic review. Author SCG will search the aforementioned databases with a reference librarian. A final list of literature after the search will be assimilated into Covidence. Duplicate records will be removed prior to the screening phase.

Two reviewers will independently screen titles and abstracts for inclusion into the full text screening phase, using the predefined inclusion and exclusion criteria. A third reviewer will resolve discrepancies between reviewers in the screening phase. During the full text screening phase, two independent reviewers will screen texts for inclusion into the study using the predefined inclusion and exclusion criteria. In studies where it is not possible to obtain the full text, the researchers will be contacted in order to request the information necessary to carry out the final inclusion process. A third reviewer will resolve discrepancies between reviewers. The main reviewer (SCG) will subsequently perform in depth evaluation of each selected full text article for inclusion into the synthesis and review.

Data Extraction:
Data will be extracted into an Excel spreadsheet utilizing standardized forms with preset variables. Data items will include but not be limited to first author, year published, years conducted, country, world bank development index, study design, control group, number of participants, statistical tests, outcomes.

Risk of bias in individual studies:
The Cochrane Grading of Recommendations Assessment, Development, and Evaluation (GRADE) approach and risk of bias tool will be utilized to assess the strength of evidence and assess the risk of bias, which covers: sequence generation, allocation concealment, binding, incomplete outcome data, and selective outcome reporting. For cohort studies, we will use the Newcastle Ottawa Scale. Two reviewers will independently assess the risk of bias of all included studies. A third reviewer will resolve discrepancies between reviewers.

Data Synthesis:
Based on the initial literature review and perceived quality of evidence, we anticipate that the data will be qualitatively synthesized. Should we find objective information to the contrary, quantitative synthesis will be considered and performed.

Roles of team members:

Reviewers SG, CB, PR, MS, and GO will serve as reviewers for title and abstract screening and for full text screening. Several other team members (IT, TB, SK) will serve in advisory and mentor roles, as is the tradition of the GEMLR group and help resolve discrepancies between reviewers in the screening phase.

Assessment of potential ethical or other risks, limitations and/or difficulties

Study environments are challenging in LMICs, especially during infectious disease outbreaks in resourceconstrained settings. Thus, the choice was made to include pre/post studies without formal controls recognizing that this will potentially influence the overall quality of the entire systematic review, particularly the synthesis of included studies. This is a limitation of the systematic review that will permit the authors to highlight cutting edge innovations in challenging environments, and it may be ameliorated by considering these studies for analysis separately of those at lower risk for bias based on the GRADE criteria.

Appendix S2

PubMed, Global Health, and Global Index Medicus databases were searched using a combination of controlled vocabulary and text word search terms, combining the concepts of severe acute respiratory infections, emergency care interventions, and low/middle-income countries as defined by World Bank Classification. Searches were conducted on November 30 2020 and January 21 2021. The results were exported to EndNote 20, and deduplicated prior to uploading the resulting reference set to Covidence for screening.

Search Strategy:

**Name of Search**	Impact of Emergency Care Interventions on Severe Acute Respiratory Infection Outcomes in Low- and Middle-Income Countries
**Databases Searched**	PubMed/MEDLINE, Global Health, Global Index Medicus
**Inclusion Criteria**	*Population*: Patients with severe acute respiratory infections in LMICs (World Bank Classification), pediatric and adult<br>*Intervention*: Emergency care interventions (medications, respiratory support, supportive care, triage, monitoring)<br>*Study type*: RCTs, observational studies with control group, observational studies with pre/post design<br>*Language*: English, Spanish
Exclusion Criteria	Population: Patients in high income countries, neonates, patients with chronic respiratory infections, patients with mild respiratory infections not requiring hospitalization, patients with sepsis or ARDS not due to a respiratory infection.<br>Intervention: Educational interventions, diagnostic tools, chronic disease management, preventative care, severity scores, prognostic scores<br>Study type: Case reports, case series, opinion papers, descriptive papers, abstracts
Concept 1	Severe acute respiratory infections (SARI)
Concept 2	Emergency care interventions
Concept 3	RCTs, observational studies with control group or pre/post design
Concept 4	Low and middle income countries

Database	PubMed/MEDLINE
Platform	PubMed
Years Searched	1940s – Present
Date Last Searched	November 30, 2020
Limits	None
Number of Results	17,286
Notes	

#1	("acute respiratory distress syndrome"[Text Word] OR "adenoviridae infections"[MeSH Terms] OR "bronchiolitis"[Text Word] OR "bronchitis"[Text Word] OR "bronchopneumonia"[Text Word] OR "coronavirus infections"[MeSH Terms] OR "coronavirus"[Text Word] OR "covid 19"[Supplementary Concept] OR "covid*"[Text Word] OR "croup"[Text Word] OR "epiglottitis"[Text Word] OR "flu"[Text Word] OR "h1n1"[Text Word] OR "influenza"[Text Word] OR "laryngitis"[Text Word] OR "laryngotracheobronchitis"[Text Word] OR "mers virus"[Text Word] OR "mers cov"[Text Word] OR "middle east respiratory syndrome"[Text Word] OR "ncov*"[Text Word] OR "orthomyxoviridae infections"[MeSH Terms] OR "picornaviridae infections"[MeSH Terms] OR "pleurisy"[Text Word] OR "pleuropneumonia"[Text Word] OR "pneumonia"[Text Word] OR "pneumovirus infections"[MeSH Terms] OR "respiratory depression"[Text Word] OR "respiratory failure"[Text Word] OR "respiratory infection*"[Text Word] OR ("respiratory insufficiency"[MeSH Terms] OR "pulmonary valve insufficiency"[MeSH Terms]) OR "respiratory insufficiency"[Text Word] OR "respiratory syncytial virus infections"[MeSH Terms] OR "respiratory syncytial virus"[Text Word] OR "respiratory tract infection*"[Text Word] OR "respiratory tract infections"[MeSH Terms] OR "respiratory viral infections"[Text Word] OR "rhinitis"[Text Word] OR "rsv"[Text Word] OR "sars cov2"[Text Word] OR "severe acute respiratory infection*"[Text Word] OR "severe acute respiratory syndrome coronavirus 2"[Supplementary Concept] OR "tuburculosis"[Text Word] OR "whooping cough"[Text Word] OR "mycoplasma pneumoniae"[Text Word] OR "pleural effusion"[Text Word] OR "pertussis"[Text Word] OR "parapneumonic effusion"[Text Word] OR "hypoxia"[Text Word] OR "hypoxemia"[Text Word] OR "respiratory distress"[Text Word] OR "pulmonary abscess"[Text Word] OR "wheezing"[Text Word] OR "stridor"[Text Word] OR "crackles"[Text Word] OR "rhonchi"[Text Word] OR "rales"[Text Word] OR "empyema"[Text Word] OR "pyothorax"[Text Word])

#2	("levalbuterol"[MeSH Terms] OR "albuterol"[MeSH Terms] OR "albuterol"[Text Word] OR "anti biotic"[Text Word] OR "anti infective agents"[MeSH Terms] OR "anti infective"[Text Word] OR "anti viral"[Text Word] OR "antibiotic"[Text Word] OR "antiviral agents"[MeSH Terms] OR "antiviral agents"[Supplementary Concept] OR "antiviral"[Text Word] OR "artificial respiration"[Text Word] OR "atomizer"[Text Word] OR "continuous monitoring"[Text Word] OR "continuous positive airway pressure"[Text Word] OR "corticosteroids"[Text Word] OR "cpap"[Text Word] OR "drug"[Text Word] OR "face down"[Text Word] OR "face down"[Text Word] OR "inhaler"[Text Word] OR "intubation, intratracheal"[MeSH Terms] OR "mechanical ventilation"[Text Word] OR "medication"[Text Word] OR "micronutrients"[MeSH Terms] OR "monitoring, physiologic"[MeSH Terms] OR "nebulizer"[Text Word] OR "nebulizers and vaporizers"[MeSH Terms] OR "non invasive ventilation"[Text Word] OR "noninvasive ventilation"[Text Word] OR "oximetry"[MeSH Terms] OR "oxygen/therapeutic use"[MeSH Terms] OR "point of care systems"[MeSH Terms] OR "position*"[Text Word] OR "postur*"[Text Word] OR "posture"[MeSH Terms] OR "prone"[Text Word] OR "pulse oximetry"[Text Word] OR "respiratory support"[Text Word] OR "respiratory system agents"[MeSH Terms] OR "respiratory therapy"[MeSH Terms] OR "risk stratification"[Text Word] OR "risk stratify"[Text Word] OR "side lying"[Text Word] OR "side lying"[Text Word] OR "side lying"[Text Word] OR "steroid"[Text Word] OR "supine"[Text Word] OR "therapeutic"[Text Word] OR "therapeutics"[MeSH Terms] OR "treat"[Text Word] OR "treatment"[Text Word] OR "upright"[Text Word] OR "vaporizer"[Text Word] OR "zinc"[Text Word] OR "vitamin"[Text Word] OR "nasal cannula"[Text Word] OR "oxygen therapy"[Text Word] OR "continuous positive airway pressure"[MeSH Terms] OR "oxygen inhalation therapy"[MeSH Terms] OR "adjuvant"[Text Word] OR "supportive care"[Text Word] OR "prehospital"[Text Word] OR "pre hospital"[Text Word] OR "salbutamol"[Text Word] OR "blow by"[Text Word] OR "face mask"[Text Word] OR "oxygen tent"[Text Word] OR "bag valve mask"[Text Word] OR "bag valve mask"[Text Word] OR "supplemental oxygen"[Text Word] OR "acute care"[Text Word] OR "critical care"[MeSH Terms] OR "critical care"[Text Word] OR "emergencies"[MeSH Terms] OR "emergencies"[Text Word] OR "emergency medical services"[MeSH Terms] OR "emergency medicine"[MeSH Terms] OR "emergency treatment"[MeSH Terms] OR "emergency"[Text Word] OR "emergicenters"[Text Word] OR "evidence based emergency medicine"[MeSH Terms] OR "triage"[Text Word])
#3	"case control"[Title/Abstract] OR "case comparison"[Title/Abstract] OR "case controlled"[Title/Abstract] OR "case referent"[Title/Abstract] OR "case comparison"[Title/Abstract] OR "case control studies"[MeSH Terms] OR "controlled clinical trial"[Publication Type] OR "epidemiologic studies"[MeSH Terms] OR "observational study"[Publication Type] OR "placebo"[Title/Abstract] OR "randomised"[Text Word] OR "randomized controlled trial"[Publication Type] OR "randomized"[Title/Abstract] OR "retrospective"[Title/Abstract] OR "randomized controlled trials as topic"[MeSH Terms] OR "multicenter study"[Publication Type] OR "multicenter studies as topic"[MeSH Terms] OR "observational studies as topic"[MeSH Terms]

#4	("developing countries"[MeSH Terms] OR "africa"[MeSH Terms] OR "asia"[MeSH Terms:noexp] OR "asia, central"[MeSH Terms] OR "asia, southeastern"[MeSH Terms] OR "asia, western"[MeSH Terms] OR "caribbean region"[MeSH Terms] OR "south america"[MeSH Terms] OR "latin america"[MeSH Terms] OR "central america"[MeSH Terms] OR "afghanistan"[MeSH Terms] OR "albania"[MeSH Terms] OR "algeria"[MeSH Terms] OR "american samoa"[MeSH Terms] OR "angola"[MeSH Terms] OR "antigua and barbuda"[MeSH Terms] OR "argentina"[MeSH Terms] OR "armenia"[MeSH Terms] OR "azerbaijan"[MeSH Terms] OR "bahrain"[MeSH Terms] OR "bangladesh"[MeSH Terms] OR "barbados"[MeSH Terms] OR "benin"[MeSH Terms] OR "republic of belarus"[MeSH Terms] OR "belize"[MeSH Terms] OR "bhutan"[MeSH Terms] OR "bolivia"[MeSH Terms] OR "bosnia and herzegovina"[MeSH Terms] OR "botswana"[MeSH Terms] OR "brazil"[MeSH Terms] OR "bulgaria"[MeSH Terms] OR "burkina faso"[MeSH Terms] OR "burundi"[MeSH Terms] OR "cambodia"[MeSH Terms] OR "cameroon"[MeSH Terms] OR "cabo verde"[MeSH Terms] OR "central african republic"[MeSH Terms] OR "chad"[MeSH Terms] OR "chile"[MeSH Terms] OR "china"[MeSH Terms] OR "colombia"[MeSH Terms] OR "comoros"[MeSH Terms] OR "congo"[MeSH Terms] OR "costa rica"[MeSH Terms] OR "cote d ivoire"[MeSH Terms] OR "croatia"[MeSH Terms] OR "cuba"[MeSH Terms] OR "cyprus"[MeSH Terms] OR "czechoslovakia"[MeSH Terms] OR "czech republic"[MeSH Terms] OR "slovakia"[MeSH Terms] OR "djibouti"[MeSH Terms] OR "democratic republic of the congo"[MeSH Terms] OR "dominica"[MeSH Terms] OR "dominican republic"[MeSH Terms] OR "timor leste"[MeSH Terms] OR "ecuador"[MeSH Terms] OR "egypt"[MeSH Terms] OR "el salvador"[MeSH Terms] OR "eritrea"[MeSH Terms] OR "estonia"[MeSH Terms] OR "ethiopia"[MeSH Terms] OR "fiji"[MeSH Terms] OR "gabon"[MeSH Terms] OR "gambia"[MeSH Terms] OR "georgia republic"[MeSH Terms] OR "ghana"[MeSH Terms] OR "greece"[MeSH Terms] OR "grenada"[MeSH Terms] OR "guatemala"[MeSH Terms] OR "guinea"[MeSH Terms] OR "guinea bissau"[MeSH Terms] OR "guam"[MeSH Terms] OR "guyana"[MeSH Terms] OR "haiti"[MeSH Terms] OR "honduras"[MeSH Terms] OR "hungary"[MeSH Terms] OR "india"[MeSH Terms] OR "indonesia"[MeSH Terms] OR "iran"[MeSH Terms] OR "iraq"[MeSH Terms] OR "jamaica"[MeSH Terms] OR "jordan"[MeSH Terms] OR "kazakhstan"[MeSH Terms] OR "kenya"[MeSH Terms] OR "korea"[MeSH Terms] OR "kosovo"[MeSH Terms] OR "kyrgyzstan"[MeSH Terms] OR "laos"[MeSH

Terms] OR "latvia"[MeSH Terms] OR "lebanon"[MeSH Terms] OR "lesotho"[MeSH Terms] OR "liberia"[MeSH Terms] OR "libya"[MeSH Terms] OR "lithuania"[MeSH Terms] OR "republic of north macedonia"[MeSH Terms] OR "madagascar"[MeSH Terms] OR "malaysia"[MeSH Terms] OR "malawi"[MeSH Terms] OR "mali"[MeSH Terms] OR "malta"[MeSH Terms] OR "mauritania"[MeSH Terms] OR "mauritius"[MeSH Terms] OR "mexico"[MeSH Terms] OR "micronesia"[MeSH Terms] OR "middle east"[MeSH Terms] OR "moldova"[MeSH Terms] OR "mongolia"[MeSH Terms] OR "montenegro"[MeSH Terms] OR "morocco"[MeSH Terms] OR "mozambique"[MeSH Terms] OR "myanmar"[MeSH Terms] OR "namibia"[MeSH Terms] OR "nepal"[MeSH Terms] OR "netherlands antilles"[MeSH Terms] OR "new caledonia"[MeSH Terms] OR "nicaragua"[MeSH Terms] OR "niger"[MeSH Terms] OR "nigeria"[MeSH Terms] OR "oman"[MeSH Terms] OR "pakistan"[MeSH Terms] OR "palau"[MeSH Terms] OR "panama"[MeSH Terms] OR "papua new guinea"[MeSH Terms] OR "paraguay"[MeSH Terms] OR "peru"[MeSH Terms] OR "philippines"[MeSH Terms] OR "poland"[MeSH Terms] OR "portugal"[MeSH Terms] OR "puerto rico"[MeSH Terms] OR "romania"[MeSH Terms] OR "russia"[MeSH Terms] OR "russia pre 1917"[MeSH Terms] OR "rwanda"[MeSH Terms] OR "saint kitts and nevis"[MeSH Terms] OR "saint lucia"[MeSH Terms] OR "saint vincent and the grenadines"[MeSH Terms] OR "samoa"[MeSH Terms] OR "saudi arabia"[MeSH Terms] OR "senegal"[MeSH Terms] OR "serbia"[MeSH Terms] OR "montenegro"[MeSH Terms] OR "seychelles"[MeSH Terms] OR "sierra leone"[MeSH Terms] OR "slovenia"[MeSH Terms] OR "sri lanka"[MeSH Terms] OR "somalia"[MeSH Terms] OR "south africa"[MeSH Terms] OR "sudan"[MeSH Terms] OR "suriname"[MeSH Terms] OR "eswatini"[MeSH Terms] OR "syria"[MeSH Terms] OR "tajikistan"[MeSH Terms] OR "tanzania"[MeSH Terms] OR "thailand"[MeSH Terms] OR "togo"[MeSH Terms] OR "tonga"[MeSH Terms] OR "trinidad and tobago"[MeSH Terms] OR "tunisia"[MeSH Terms] OR "turkey"[MeSH Terms] OR "turkmenistan"[MeSH Terms] OR "uganda"[MeSH Terms] OR "ukraine"[MeSH Terms] OR "uruguay"[MeSH Terms] OR "ussr"[MeSH Terms] OR "uzbekistan"[MeSH Terms] OR "vanuatu"[MeSH Terms] OR "venezuela"[MeSH Terms] OR "vietnam"[MeSH Terms] OR "yemen"[MeSH Terms] OR "yugoslavia"[MeSH Terms] OR "zambia"[MeSH Terms] OR "zimbabwe"[MeSH Terms] OR "developing country"[Text Word] OR "developing countries"[Text Word] OR "developing nation"[Text Word] OR "developing nations"[Text Word] OR "developing population"[Text Word] OR "developing populations"[Text Word] OR "developing world"[Text Word] OR "less developed country"[Text Word] OR "less developed countries"[Text Word] OR "less developed nation"[Text Word] OR "less developed nations"[Text Word] OR "less developed world"[Text Word] OR "lesser developed countries"[Text Word] OR "lesser developed nations"[Text Word] OR "least developed country"[Text Word] OR "least developed countries"[Text Word] OR "least developed nations"[Text Word] OR "under developed country"[Text Word] OR "under developed countries"[Text Word] OR "under developed nations"[Text Word] OR "under developed world"[Text Word] OR "underdeveloped country"[Text Word] OR "underdeveloped countries"[Text Word] OR "underdeveloped nation"[Text Word]

OR "underdeveloped nations"[Text Word] OR "underdeveloped population"[Text Word] OR "underdeveloped populations"[Text Word] OR "underdeveloped world"[Text Word] OR "middle income country"[Text Word] OR "middle income countries"[Text Word] OR "middle income nation"[Text Word] OR "middle income nations"[Text Word] OR "middle income population"[Text Word] OR "middle income populations"[Text Word] OR "low income country"[Text Word] OR "low income countries"[Text Word] OR "low income nation"[Text Word] OR "low income nations"[Text Word] OR "low income population"[Text Word] OR "low income populations"[Text Word] OR "lower income country"[Text Word] OR "lower income countries"[Text Word] OR "lower income nations"[Text Word] OR "lower income population"[Text Word] OR "lower income populations"[Text Word] OR "underserved countries"[Text Word] OR "underserved nations"[Text Word] OR "underserved population"[Text Word] OR "underserved populations"[Text Word] OR "under served population"[Text Word] OR "under served populations"[Text Word] OR "deprived countries"[Text Word] OR "deprived population"[Text Word] OR "deprived populations"[Text Word] OR "poor country"[Text Word] OR "poor countries"[Text Word] OR "poor nation"[Text Word] OR "poor nations"[Text Word] OR "poor population"[Text Word] OR "poor populations"[Text Word] OR "poor world"[Text Word] OR "poorer countries"[Text Word] OR "poorer nations"[Text Word] OR "poorer population"[Text Word] OR "poorer populations"[Text Word] OR "developing economy"[Text Word] OR "developing economies"[Text Word] OR "less developed economy"[Text Word] OR "less developed economies"[Text Word] OR "underdeveloped economies"[Text Word] OR "middle income economy"[Text Word] OR "middle income economies"[Text Word] OR "low income economy"[Text Word] OR "low income economies"[Text Word] OR "lower income economies"[Text Word] OR "low gdp"[Text Word] OR "low gnp"[Text Word] OR "low gross domestic"[Text Word] OR "low gross national"[Text Word] OR "lower gdp"[Text Word] OR "lower gross domestic"[Text Word] OR "lmic"[Text Word] OR "lmics"[Text Word] OR "third world"[Text Word] OR "lami country"[Text Word] OR "lami countries"[Text Word] OR "transitional country"[Text Word] OR "transitional countries"[Text Word] OR "africa"[Text Word] OR "asia"[Text Word] OR "west indies"[Text Word] OR "south america"[Text Word] OR "latin america"[Text Word] OR "central america"[Text Word] OR "afghanistan"[Text Word] OR "albania"[Text Word] OR "algeria"[Text Word] OR "angola"[Text Word] OR "antigua"[Text Word] OR "barbuda"[Text Word] OR "argentina"[Text Word] OR "armenia"[Text Word] OR "armenian"[Text Word] OR "aruba"[Text Word] OR "azerbaijan"[Text Word] OR "bahrain"[Text Word] OR "bangladesh"[Text Word] OR "barbados"[Text Word] OR "benin"[Text Word] OR "byelarus"[Text Word] OR "byelorussian"[Text Word] OR "belarus"[Text Word] OR "belorussian"[Text Word] OR "belorussia"[Text Word] OR "belize"[Text Word] OR "bhutan"[Text Word] OR "bolivia"[Text Word] OR "bosnia"[Text Word] OR "herzegovina"[Text Word] OR "hercegovina"[Text Word] OR "botswana"[Text Word] OR "brasil"[Text Word] OR "brazil"[Text Word] OR "bulgaria"[Text Word] OR "burkina faso"[Text Word] OR "burkina fasso"[Text Word] OR "upper volta"[Text Word] OR "burundi"[Text Word] OR "urundi"[Text

Word] OR "cambodia"[Text Word] OR "khmer republic"[Text Word] OR "kampuchea"[Text Word] OR "cameroon"[Text Word] OR "cameroons"[Text Word] OR "cameron"[Text Word] OR "cape verde"[Text Word] OR "central african republic"[Text Word] OR "chad"[Text Word] OR "chile"[Text Word] OR "china"[Text Word] OR "colombia"[Text Word] OR "comoros"[Text Word] OR "comoro islands"[Text Word] OR "comores"[Text Word] OR "mayotte"[Text Word] OR "congo"[Text Word] OR "zaire"[Text Word] OR "costa rica"[Text Word] OR "cote d ivoire"[Text Word] OR "ivory coast"[Text Word] OR "croatia"[Text Word] OR "cuba"[Text Word] OR "cyprus"[Text Word] OR "czechoslovakia"[Text Word] OR "czech republic"[Text Word] OR "slovakia"[Text Word] OR "slovak republic"[Text Word] OR "djibouti"[Text Word] OR "french somaliland"[Text Word] OR "dominica"[Text Word] OR "dominican republic"[Text Word] OR "east timor"[Text Word] OR "timor leste"[Text Word] OR "ecuador"[Text Word] OR "egypt"[Text Word] OR "united arab republic"[Text Word] OR "el salvador"[Text Word] OR "eritrea"[Text Word] OR "estonia"[Text Word] OR "ethiopia"[Text Word] OR "fiji"[Text Word] OR "gabon"[Text Word] OR "gabonese republic"[Text Word] OR "gambia"[Text Word] OR "gaza"[Text Word] OR "georgia republic"[Text Word] OR "georgian republic"[Text Word] OR "ghana"[Text Word] OR "gold coast"[Text Word] OR "greece"[Text Word] OR "grenada"[Text Word] OR "guatemala"[Text Word] OR "guinea"[Text Word] OR "guam"[Text Word] OR "guiana"[Text Word] OR "guyana"[Text Word] OR "haiti"[Text Word] OR "honduras"[Text Word] OR "hungary"[Text Word] OR "india"[Text Word] OR "maldives"[Text Word] OR "indonesia"[Text Word] OR "iran"[Text Word] OR "iraq"[Text Word] OR "isle of man"[Text Word] OR "jamaica"[Text Word] OR "jordan"[Text Word] OR "kazakhstan"[Text Word] OR "kazakh"[Text Word] OR "kenya"[Text Word] OR "kiribati"[Text Word] OR "korea"[Text Word] OR "kosovo"[Text Word] OR "kyrgyzstan"[Text Word] OR "kirghizia"[Text Word] OR "kyrgyz republic"[Text Word] OR "kirghiz"[Text Word] OR "kirgizstan"[Text Word] OR "lao pdr"[Text Word] OR "laos"[Text Word] OR "latvia"[Text Word] OR "lebanon"[Text Word] OR "lesotho"[Text Word] OR "basutoland"[Text Word] OR "liberia"[Text Word] OR "libya"[Text Word] OR "lithuania"[Text Word] OR "macedonia"[Text Word] OR "madagascar"[Text Word] OR "malagasy republic"[Text Word] OR "malaysia"[Text Word] OR "malaya"[Text Word] OR "malay"[Text Word] OR "sabah"[Text Word] OR "sarawak"[Text Word] OR "malawi"[Text Word] OR "nyasaland"[Text Word] OR "mali"[Text Word] OR "malta"[Text Word] OR "marshall islands"[Text Word] OR "mauritania"[Text Word] OR "mauritius"[Text Word] OR "agalega islands"[Text Word] OR "mexico"[Text Word] OR "micronesia"[Text Word] OR "moldova"[Text Word] OR "moldovia"[Text Word] OR "moldovian"[Text Word] OR "mongolia"[Text Word] OR "montenegro"[Text Word] OR "morocco"[Text Word] OR "ifni"[Text Word] OR "mozambique"[Text Word] OR "myanmar"[Text Word] OR "myanma"[Text Word] OR "burma"[Text Word] OR "namibia"[Text Word] OR "nepal"[Text Word] OR "netherlands antilles"[Text Word] OR "new caledonia"[Text Word] OR "nicaragua"[Text Word] OR "niger"[Text Word] OR "nigeria"[Text Word] OR "northern mariana

islands"[Text Word] OR "oman"[Text Word] OR "muscat"[Text Word] OR "pakistan"[Text Word] OR "palau"[Text Word] OR "palestine"[Text Word] OR "panama"[Text Word] OR "paraguay"[Text Word] OR "peru"[Text Word] OR "philippines"[Text Word] OR "philipines"[Text Word] OR "phillipines"[Text Word] OR "phillippines"[Text Word] OR "poland"[Text Word] OR "portugal"[Text Word] OR "puerto rico"[Text Word] OR "rhodesia"[Text Word] OR "romania"[Text Word] OR "rumania"[Text Word] OR "roumania"[Text Word] OR "russia"[Text Word] OR "russian"[Text Word] OR "rwanda"[Text Word] OR "ruanda"[Text Word] OR "saint kitts"[Text Word] OR "st kitts"[Text Word] OR "nevis"[Text Word] OR "saint lucia"[Text Word] OR "st lucia"[Text Word] OR "saint vincent"[Text Word] OR "st vincent"[Text Word] OR "grenadines"[Text Word] OR "samoa"[Text Word] OR "samoan islands"[Text Word] OR "sao tome"[Text Word] OR "saudi arabia"[Text Word] OR "senegal"[Text Word] OR "serbia"[Text Word] OR "montenegro"[Text Word] OR "seychelles"[Text Word] OR "sierra leone"[Text Word] OR "slovenia"[Text Word] OR "sri lanka"[Text Word] OR "ceylon"[Text Word] OR "solomon islands"[Text Word] OR

	"somalia"[Text Word] OR "sudan"[Text Word] OR "suriname"[Text Word] OR "surinam"[Text Word] OR "swaziland"[Text Word] OR "syria"[Text Word] OR "tajikistan"[Text Word] OR "tadzhikistan"[Text Word] OR "tadjikistan"[Text Word] OR "tadzhik"[Text Word] OR "tanzania"[Text Word] OR "thailand"[Text Word] OR "togo"[Text Word] OR "togolese republic"[Text Word] OR "tonga"[Text Word] OR "trinidad"[Text Word] OR "tobago"[Text Word] OR "tunisia"[Text Word] OR "turkey"[Text Word] OR "turkmenistan"[Text Word] OR "turkmen"[Text Word] OR "uganda"[Text Word] OR "ukraine"[Text Word] OR "uruguay"[Text Word] OR "ussr"[Text Word] OR "soviet union"[Text Word] OR "union of soviet socialist republics"[Text Word] OR "uzbekistan"[Text Word] OR "vanuatu"[Text Word] OR "new hebrides"[Text Word] OR "venezuela"[Text Word] OR "vietnam"[Text Word] OR "viet nam"[Text Word] OR "west bank"[Text Word] OR "yemen"[Text Word] OR "yugoslavia"[Text Word] OR "zambia"[Text Word] OR "zimbabwe"[Text Word])	
#5	Search #1 AND #2 AND #3 AND #4	17,286

Database	Global Health
Platform	Ebsco
Years Searched	1973-present
Date Last Searched	January 21, 2021
Limits	None
Number of Results	4,506
Notes	Altered terms to remove respiratory failure, respiratory insufficiency, respiratory depression

TX ("Acute Respiratory Distress Syndrome" OR "Adenoviridae Infections" OR "Bronchiolitis" OR "Bronchitis" OR "Bronchopneumonia" OR "Coronavirus Infections" OR "coronavirus" OR "COVID-19 " OR "covid" OR "crackles" OR "croup" OR "empyema" OR "epiglottitis" OR "flu" OR "h1n1" OR "Influenza" OR "laryngitis" OR "laryngotracheobronchitis" OR "MERS Virus" OR "MERS-CoV" OR "Middle East respiratory syndrome" OR "mycoplasma pneumoniae" OR "nCov" OR "Orthomyxoviridae Infections" OR "parapneumonic effusion" OR #1

"pertussis" OR "Picornaviridae Infections" OR "pleural effusion" OR "pleurisy" OR "pleuropneumonia" OR "pneumonia" OR "Pneumovirus Infections" OR "pulmonary abscess" OR "pyothorax" OR "rales" OR "respiratory infection" OR "Respiratory Syncytial Virus" OR "respiratory tract infection" OR "respiratory viral infection" OR "rhinitis" OR "rhonchi" OR "RSV" OR "sars-cov2" OR "severe acute respiratory infection" OR "severe acute respiratory syndrome coronavirus 2" OR "stridor" OR "tuburculosis" OR "wheezing" OR "whooping cough")

TX ("acute care" OR "adjuvant" OR "Albuterol" OR "albuterol" OR "anti-biotic" OR "anti-infective" OR "anti-viral" OR "antibiotic" OR "antiviral" OR "artificial respiration" OR "atomizer" OR "bag valve mask" OR "bag-valve mask" OR "blow by" OR "continuous monitoring" OR "continuous positive airway pressure" OR #2

"corticosteroids" OR "CPAP" OR "Critical Care" OR "drug" OR "Emergencies" OR "Emergency Medical Services" OR "Emergency Medicine" OR "Emergency Treatment" OR "emergency" OR "Emergicenters" OR "face down" OR "face mask" OR "face-down" OR "inhaler" OR "intubation" OR "mechanical ventilation" OR

"medication" OR "Micronutrients" OR "nasal cannula" OR "nebulizer" OR "noninvasive ventilation" OR "noninvasive ventilation" OR "Oximetry" OR "Oxygen Inhalation Therapy" OR "oxygen tent" OR "oxygen therapy" OR "positioning" OR "Posture" OR "pre-hospital" OR "prehospital" OR "prone" OR "pulse oximetry" OR "Respiratory support" OR "Respiratory System Agents" OR "Respiratory therapy" OR "risk stratification" OR "risk-stratify" OR "salbutamol" OR "side lying" OR "side-lying" OR "steroid" OR "supine" OR "supplemental oxygen" OR "supportive care" OR "therapeutic" OR "triage" OR "upright" OR "vaporizer" OR "vitamin" OR "zinc")

#3 TX ("Multicenter Study" OR "case comparison" OR "case control" OR "case controlled" OR "case referent" OR "Case-Comparison" OR "controlled clinical trial" OR "Observational Study " OR "Placebo" OR "randomised" OR "randomized" OR "randomized controlled trial" OR "retrospective")

TX (afghanistan OR albania OR algeria OR "american samoa" OR angola OR "antigua and barbuda" OR antigua OR barbuda OR argentina OR armenia OR armenian OR aruba OR azerbaijan OR bahrain OR bangladesh OR barbados OR "republic of belarus" OR belarus OR byelarus OR belorussia OR byelorussian OR belize OR "british honduras" OR benin OR dahomey OR bhutan OR bolivia OR "bosnia and herzegovina" OR bosnia OR herzegovina OR botswana OR bechuanaland OR brazil OR brasil OR bulgaria OR "burkina faso" OR "burkina fasso" OR "upper volta" OR burundi OR urundi OR "cabo verde" OR "cape verde" OR cambodia OR kampuchea OR "khmer republic" OR cameroon OR cameron OR cameroun OR "central african republic" OR "ubangi shari" OR chad OR chile OR china OR colombia OR comoros OR "comoro islands" OR "iles comores" OR mayotte OR "democratic republic of the congo" OR "democratic republic congo" OR congo OR zaire OR "costa rica" OR "cote d’ivoire" OR "cote d’ ivoire" OR "cote divoire" OR "cote d ivoire" OR "ivory coast" OR croatia OR cuba OR cyprus OR "czech republic" OR czechoslovakia OR djibouti OR "french somaliland" OR dominica OR "dominican republic" OR ecuador OR egypt OR "united arab republic" OR "el salvador" OR "equatorial guinea" OR "spanish guinea" OR eritrea OR estonia OR eswatini OR swaziland OR ethiopia OR fiji OR gabon OR "gabonese republic" OR gambia OR "georgia (republic)" OR georgia OR georgian

OR ghana OR "gold coast" OR gibraltar OR greece OR grenada OR guam OR guatemala OR guinea OR "guinea bissau" OR guyana OR "british guiana" OR haiti OR hispaniola OR honduras OR hungary OR india OR indonesia OR timor OR iran OR iraq OR "isle of man" OR jamaica OR jordan OR kazakhstan OR kazakh OR kenya OR "democratic people’s republic of korea" OR "republic of korea" OR north korea OR south korea OR korea OR kosovo OR kyrgyzstan OR kirghizia OR kirgizstan OR "kyrgyz republic" OR kirghiz OR laos OR "lao pdr" OR "lao people's democratic republic" OR latvia OR lebanon OR "lebanese republic" OR lesotho OR basutoland OR liberia OR libya OR "libyan arab jamahiriya" OR lithuania OR macau OR macao OR "republic of north macedonia" OR macedonia OR madagascar OR "malagasy republic" OR malawi OR nyasaland OR malaysia OR "malay federation" OR "malaya federation" OR maldives OR "indian ocean islands" OR "indian ocean" OR mali OR malta OR micronesia OR "federated states of micronesia" OR kiribati OR "marshall islands" OR nauru OR "northern mariana islands" OR palau OR tuvalu OR mauritania OR mauritius OR mexico OR moldova OR moldovian OR mongolia OR montenegro OR morocco OR ifni OR mozambique OR "portuguese east africa" OR myanmar OR burma OR namibia OR nepal OR "netherlands antilles" OR nicaragua OR niger OR nigeria OR oman OR muscat OR

#4 pakistan OR panama OR "papua new guinea" OR paraguay OR peru

OR philippines OR philipines OR phillipines OR phillippines OR poland OR "polish people's republic" OR portugal OR "portuguese republic" OR "puerto rico" OR romania OR russia OR "russian federation" OR ussr OR "soviet union" OR "union of soviet socialist republics" OR rwanda OR ruanda OR samoa OR "pacific islands" OR polynesia OR "samoan islands" OR "navigator island" OR "navigator islands" OR "sao tome and principe" OR "saudi arabia" OR senegal OR serbia OR seychelles OR "sierra leone" OR slovakia OR "slovak republic" OR slovenia OR melanesia OR "solomon island" OR "solomon islands" OR "norfolk island" OR "norfolk islands" OR somalia OR "south africa" OR "south sudan" OR "sri lanka" OR ceylon OR "saint kitts and nevis" OR "st. kitts and nevis" OR "saint lucia" OR "st. lucia" OR "saint vincent and the grenadines" OR "saint vincent" OR "st. vincent" OR grenadines OR sudan OR suriname OR surinam OR "dutch guiana" OR "netherlands guiana" OR syria OR "syrian arab republic" OR tajikistan OR tadjikistan OR tadzhikistan OR tadzhik OR tanzania OR tanganyika OR thailand OR siam OR "timor leste" OR "east timor" OR togo OR "togolese republic" OR tonga OR "trinidad and tobago" OR trinidad OR tobago OR tunisia OR turkey OR turkmenistan OR turkmen OR uganda OR ukraine OR uruguay OR uzbekistan OR uzbek OR vanuatu OR "new hebrides" OR venezuela OR vietnam OR "viet nam" OR "middle east" OR "west bank" OR gaza OR palestine OR yemen OR yugoslavia OR zambia OR zimbabwe OR "northern rhodesia" OR "global south" OR "africa south of the sahara" OR "sub saharan africa" OR "subsaharan africa" OR "africa, central" OR "central africa" OR "africa, northern" OR "north africa" OR "northern africa" OR magreb OR maghrib OR sahara OR "africa, southern" OR "southern africa" OR "africa, eastern" OR "east africa" OR "eastern africa" OR "africa, western" OR "west africa" OR "western africa" OR "west indies" OR "indian ocean islands" OR caribbean OR "central america" OR "latin america" OR "south and central america" OR "south america" OR "asia, central" OR "central asia" OR "asia, northern" OR "north asia" OR "northern asia" OR "asia, southeastern" OR "southeastern asia" OR "south eastern asia" OR "southeast asia" OR "south east asia" OR "asia, western" OR "western asia" OR "europe, eastern" OR "east europe"
OR "eastern europe" OR "developing country" OR "developing countries" OR "developing nation" OR "developing nations" OR "developing population" OR "developing populations" OR "developing world" OR "less developed country" OR "less developed countries" OR "less developed nation" OR "less developed nations" OR "less developed population" OR "less developed populations" OR "less developed world" OR "lesser developed country" OR "lesser developed countries" OR "lesser developed nation" OR "lesser developed nations" OR "lesser developed population" OR "lesser developed populations" OR "lesser developed world" OR "under developed country" OR "under developed countries" OR "under developed nation" OR "under developed nations" OR "under developed population" OR "under developed populations" OR "under developed world" OR "underdeveloped country" OR "underdeveloped countries" OR "underdeveloped nation" OR "underdeveloped nations" OR "underdeveloped population" OR "underdeveloped populations" OR "underdeveloped world" OR "middle income country" OR "middle income countries" OR "middle income nation" OR "middle income nations" OR "middle income population" OR "middle income populations" OR "low income country" OR "low income countries" OR "low income nation" OR "low income nations" OR "low income population" OR "low income populations" OR "lower income country" OR "lower income countries" OR "lower income nation" OR "lower income nations" OR "lower income population" OR "lower income populations" OR "underserved country" OR "underserved countries" OR "underserved nation"

	OR "underserved nations" OR "underserved population" OR "underserved populations" OR "underserved world" OR "under served country" OR "under served countries" OR "under served nation" OR "under served nations" OR "under served population" OR "under served populations" OR "under served world" OR "deprived country" OR "deprived countries" OR "deprived nation" OR "deprived nations" OR "deprived population" OR "deprived populations" OR "deprived world" OR "poor country" OR "poor countries" OR "poor nation" OR "poor nations" OR "poor population" OR "poor populations" OR "poor world" OR "poorer country" OR "poorer countries" OR "poorer nation" OR "poorer nations" OR "poorer population" OR "poorer populations" OR "poorer world" OR "developing economy" OR "developing economies" OR "less developed economy" OR "less developed economies" OR "lesser developed economy" OR "lesser developed economies" OR "under developed economy" OR "under developed economies" OR "underdeveloped economy" OR "underdeveloped economies" OR "middle income economy" OR "middle income economies" OR "low income economy" OR "low income economies" OR "lower income economy" OR "lower income economies" OR "low gdp" OR "low gnp" OR "low gross domestic" OR "low gross national" OR "lower gdp" OR "lower gnp" OR "lower gross domestic" OR "lower gross national" OR lmic OR lmics OR "third world" OR "lami country" OR "lami countries" OR "transitional country" OR "transitional countries" OR "emerging economies" OR "emerging nation" OR "emerging nations")	
#5	Search #1 AND #2 AND #3 AND #4	4,506

Database	WHO Global Index Medicus
Platform	https://www.globalindexmedicus.net/
Years Searched	
Date Last Searched	January 21, 2021
Limits	None
Number of Results	3,388
Notes	Date coverage is not available

#1	(tw:("Acute Respiratory Distress Syndrome" OR "Adenoviridae Infections" OR "Bronchiolitis" OR "Bronchitis" OR "Bronchopneumonia" OR "Coronavirus Infections" OR "coronavirus" OR "COVID-19 " OR "covid" OR "crackles" OR "croup" OR "empyema" OR "epiglottitis" OR "flu" OR "h1n1" OR "Influenza" OR "laryngitis" OR "laryngotracheobronchitis" OR "MERS Virus" OR "MERS-CoV" OR "Middle East respiratory syndrome" OR "mycoplasma pneumoniae" OR "nCov" OR "Orthomyxoviridae Infections" OR "parapneumonic effusion" OR "pertussis" OR "Picornaviridae Infections" OR "pleural effusion" OR "pleurisy" OR "pleuropneumonia" OR "pneumonia" OR "Pneumovirus Infections" OR "pulmonary abscess" OR "pyothorax" OR "rales" OR "respiratory infection" OR "Respiratory Syncytial Virus" OR "respiratory tract infection" OR "respiratory viral infection" OR "rhinitis" OR "rhonchi" OR "RSV" OR "sars-cov2" OR "severe acute respiratory infection" OR "severe acute respiratory syndrome coronavirus 2" OR "stridor" OR "tuburculosis" OR "wheezing" OR "whooping cough")
#2	(tw:("acute care" OR "adjuvant" OR "Albuterol" OR "albuterol" OR "anti-biotic" OR "anti-infective" OR "anti-viral" OR "antibiotic" OR "antiviral" OR "artificial respiration" OR "atomizer" OR "bag valve mask" OR "bag-valve mask" OR "blow by" OR "continuous monitoring" OR "continuous positive airway pressure" OR "corticosteroids" OR "CPAP" OR "Critical Care" OR "drug" OR "Emergencies" OR "Emergency Medical Services" OR "Emergency Medicine" OR "Emergency Treatment" OR "emergency" OR "Emergicenters" OR "face down" OR "face mask" OR "face-down" OR "inhaler" OR "intubation" OR "mechanical ventilation" OR "medication" OR "Micronutrients" OR "nasal cannula" OR "nebulizer" OR "noninvasive ventilation" OR "noninvasive ventilation" OR "Oximetry" OR "Oxygen Inhalation Therapy" OR "oxygen tent" OR "oxygen therapy" OR "positioning" OR "Posture" OR "pre-hospital" OR "prehospital" OR "prone" OR "pulse oximetry" OR "Respiratory support" OR "Respiratory System Agents" OR "Respiratory therapy" OR "risk stratification" OR "risk-stratify" OR "salbutamol" OR "side lying" OR "side-lying" OR "steroid" OR "supine" OR "supplemental oxygen" OR "supportive care" OR "therapeutic" OR "triage" OR "upright" OR "vaporizer" OR "vitamin" OR "zinc")
#3	(tw:("Multicenter Study" OR "case comparison" OR "case control" OR "case controlled" OR "case referent" OR "Case-Comparison" OR "controlled clinical trial" OR "Observational Study " OR "Placebo" OR "randomised" OR "randomized" OR "randomized controlled trial" OR "retrospective")

#4	(tw:(afghanistan OR albania OR algeria OR "american samoa" OR angola OR "antigua and barbuda" OR antigua OR barbuda OR argentina OR armenia OR armenian OR aruba OR azerbaijan OR bahrain OR bangladesh OR barbados OR "republic of belarus" OR belarus OR byelarus OR belorussia OR byelorussian OR belize OR "british honduras" OR benin OR dahomey OR bhutan OR bolivia OR "bosnia and herzegovina" OR bosnia OR herzegovina OR botswana OR bechuanaland OR brazil OR brasil OR bulgaria OR "burkina faso" OR "burkina fasso" OR "upper volta" OR burundi OR urundi OR "cabo verde" OR "cape verde" OR cambodia OR kampuchea OR "khmer republic" OR cameroon OR cameron OR cameroun OR "central african republic" OR "ubangi shari" OR chad OR chile OR china OR colombia OR comoros OR "comoro islands" OR "iles comores" OR mayotte OR "democratic republic of the congo" OR "democratic republic congo" OR congo OR zaire OR "costa rica" OR "cote d'ivoire" OR "cote d' ivoire" OR "cote divoire" OR "cote d ivoire" OR "ivory coast" OR croatia OR cuba OR cyprus OR "czech republic" OR czechoslovakia OR djibouti OR "french somaliland" OR dominica OR "dominican republic" OR ecuador OR egypt OR "united arab republic" OR "el salvador" OR "equatorial guinea" OR "spanish guinea" OR eritrea OR estonia OR eswatini OR swaziland OR ethiopia OR fiji OR gabon OR "gabonese republic" OR gambia OR "georgia (republic)" OR georgia OR georgian OR ghana OR "gold coast" OR gibraltar OR greece OR grenada OR guam OR guatemala OR guinea OR "guinea bissau" OR guyana OR "british guiana" OR haiti OR hispaniola OR honduras OR hungary OR india OR indonesia OR timor OR iran OR iraq OR "isle of man" OR jamaica OR jordan OR kazakhstan OR kazakh OR kenya OR "democratic people's republic of korea" OR "republic of korea" OR north korea OR south korea OR korea OR kosovo OR kyrgyzstan OR kirghizia OR kirgizstan OR "kyrgyz republic" OR kirghiz OR laos OR "lao pdr" OR "lao people's democratic republic" OR latvia OR lebanon OR "lebanese republic" OR lesotho OR basutoland OR liberia OR libya OR "libyan arab jamahiriya" OR lithuania OR macau OR macao OR "republic of north macedonia" OR macedonia OR madagascar OR "malagasy republic" OR malawi OR nyasaland OR malaysia OR "malay federation" OR "malaya federation" OR maldives OR "indian ocean islands" OR "indian ocean" OR mali OR malta OR micronesia OR "federated states of micronesia" OR kiribati OR "marshall islands" OR nauru OR "northern mariana islands" OR palau OR tuvalu OR mauritania OR mauritius OR mexico OR moldova

OR moldovian OR mongolia OR montenegro OR morocco OR ifni OR mozambique OR "portuguese east africa" OR myanmar OR burma OR namibia OR nepal OR "netherlands antilles" OR nicaragua OR niger OR nigeria OR oman OR muscat OR pakistan OR panama OR "papua new guinea" OR paraguay OR peru OR philippines OR philipines OR phillipines OR phillippines OR poland OR
"polish people's republic" OR portugal OR "portuguese republic" OR "puerto rico" OR romania OR russia OR "russian federation" OR ussr OR "soviet union" OR "union of soviet socialist republics" OR rwanda OR ruanda OR samoa OR "pacific islands" OR polynesia OR "samoan islands" OR "navigator island" OR "navigator islands" OR "sao tome and principe" OR "saudi arabia" OR senegal OR serbia OR seychelles OR "sierra leone" OR slovakia OR "slovak republic" OR slovenia OR melanesia OR "solomon island" OR "solomon islands" OR "norfolk island" OR "norfolk islands" OR somalia OR "south africa" OR "south sudan" OR "sri lanka" OR ceylon OR "saint kitts and nevis" OR "st. kitts and nevis" OR "saint lucia" OR "st. lucia" OR "saint vincent and the grenadines" OR "saint vincent" OR "st. vincent" OR grenadines OR sudan OR suriname OR surinam OR "dutch guiana" OR "netherlands guiana" OR syria OR "syrian arab republic" OR tajikistan OR tadjikistan OR tadzhikistan OR tadzhik OR tanzania OR tanganyika OR thailand OR siam OR "timor leste" OR "east timor" OR togo OR "togolese republic" OR tonga OR "trinidad and tobago" OR trinidad OR tobago OR tunisia OR turkey OR turkmenistan OR turkmen OR uganda OR ukraine OR uruguay OR uzbekistan OR uzbek OR vanuatu OR "new hebrides" OR venezuela OR vietnam OR "viet nam" OR "middle east" OR "west bank" OR gaza OR palestine OR yemen OR yugoslavia OR zambia OR zimbabwe OR "northern rhodesia" OR "global south" OR "africa south of the sahara" OR "sub saharan africa" OR "subsaharan africa" OR "africa, central" OR "central africa" OR "africa, northern" OR "north africa" OR "northern africa" OR magreb OR maghrib OR sahara OR "africa, southern" OR "southern africa" OR "africa, eastern" OR "east africa" OR "eastern africa" OR "africa, western" OR "west africa" OR "western africa" OR "west indies" OR "indian ocean islands" OR caribbean OR "central america" OR "latin america" OR "south and central america" OR "south america" OR "asia, central" OR "central asia" OR "asia, northern" OR "north asia" OR "northern asia" OR "asia, southeastern" OR "southeastern asia" OR "south eastern asia" OR "southeast asia" OR "south east asia" OR "asia, western" OR "western asia" OR "europe, eastern" OR "east europe"
OR "eastern europe" OR "developing country" OR "developing countries" OR "developing nation" OR "developing nations" OR "developing population" OR "developing populations" OR "developing world" OR "less developed country" OR "less developed countries" OR "less developed nation" OR "less developed nations" OR "less developed population" OR "less developed populations" OR "less developed world" OR "lesser developed country" OR "lesser developed countries" OR "lesser developed nation" OR "lesser developed nations" OR "lesser developed population" OR "lesser developed populations" OR "lesser developed world" OR "under developed country" OR "under developed countries" OR "under developed nation" OR "under developed nations" OR "under developed population" OR "under developed populations" OR "under developed world" OR "underdeveloped country" OR "underdeveloped countries" OR "underdeveloped nation" OR "underdeveloped nations" OR "underdeveloped population" OR "underdeveloped populations" OR "underdeveloped world" OR "middle income country" OR "middle income countries" OR "middle income nation" OR "middle income nations" OR "middle income population" OR "middle income populations" OR "low income country" OR "low income countries" OR "low income nation" OR "low income nations" OR

	"low income population" OR "low income populations" OR "lower income country" OR "lower income countries" OR "lower income nation" OR "lower income nations" OR "lower income population" OR "lower income populations" OR "underserved country" OR "underserved countries" OR "underserved nation" OR "underserved nations" OR "underserved population" OR "underserved populations" OR "underserved world" OR "under served country" OR "under served countries" OR "under served nation" OR "under served nations" OR "under served population" OR "under served populations" OR "under served world" OR "deprived country" OR "deprived countries" OR "deprived nation" OR "deprived nations" OR "deprived population" OR "deprived populations" OR "deprived world" OR "poor country" OR "poor countries" OR "poor nation" OR "poor nations" OR "poor population" OR "poor populations" OR "poor world" OR "poorer country" OR "poorer countries" OR "poorer nation" OR "poorer nations" OR "poorer population" OR "poorer populations" OR "poorer world" OR "developing economy" OR "developing economies" OR "less developed economy" OR "less developed economies" OR "lesser developed economy" OR "lesser developed economies" OR "under developed economy" OR "under developed economies" OR "underdeveloped economy" OR "underdeveloped economies" OR "middle income economy" OR "middle income economies" OR "low income economy" OR "low income economies" OR "lower income economy" OR "lower income economies" OR "low gdp" OR "low gnp" OR "low gross domestic" OR "low gross national" OR "lower gdp" OR "lower gnp" OR "lower gross domestic" OR "lower gross national" OR lmic OR lmics OR "third world" OR "lami country" OR "lami countries" OR "transitional country" OR "transitional countries" OR "emerging economies" OR "emerging nation" OR "emerging nations")	
#5	Search #1 AND #2 AND #3 AND #4	3,388

Summary

Total references retrieved	25,180
# Duplicates removed in EndNote (prior to Covidence upload)	4,462
# Duplicates detected in Covidence:	70
Total # references added to Title/Abstract Screening	20,648
